# Differences in carbonate chemistry up-regulation of long-lived reef-building corals

**DOI:** 10.1038/s41598-023-37598-9

**Published:** 2023-07-18

**Authors:** Marine Canesi, Eric Douville, Paolo Montagna, Marco Taviani, Jarosław Stolarski, Louise Bordier, Arnaud Dapoigny, Gninwoyo Eric Hermann Coulibaly, Anne-Catherine Simon, Mathieu Agelou, Jonathan Fin, Nicolas Metzl, Guillaume Iwankow, Denis Allemand, Serge Planes, Clémentine Moulin, Fabien Lombard, Guillaume Bourdin, Romain Troublé, Sylvain Agostini, Bernard Banaigs, Emilie Boissin, Emmanuel Boss, Chris Bowler, Colomban de Vargas, Michel Flores, Didier Forcioli, Paola Furla, Eric Gilson, Pierre E. Galand, Stéphane Pesant, Shinichi Sunagawa, Olivier P. Thomas, Rebecca Vega Thurber, Christian R. Voolstra, Patrick Wincker, Didier Zoccola, Stéphanie Reynaud

**Affiliations:** 1 Laboratoire des Sciences du Climat et de l’Environnement, LSCE/IPSL, UMR 8212 CEA-CNRS-UVSQ, Université Paris-Saclay, 91191 Gif-Sur-Yvette, France; 2 Centre Scientifique de Monaco, 8 Quai Antoine Ier, 98000 Monaco City, Monaco; 3 LIA ROPSE, Laboratoire International Associé Université Côte d’Azur - Centre Scientifique de Monaco – U FR, Monaco City, Monaco; 4 Institute of Polar Sciences (ISP), CNR, Via Gobetti 101, 40129 Bologna, Italy; 5 Stazione Zoologica Anton Dohrn, Villa Comunale, 80121 Napoli, Italy; 6 Institute of Marine Sciences (ISMAR), CNR, Via Gobetti 101, 40129 Bologna, Italy; 7 Institute of Paleobiology, Polish Academy of Sciences, 00818 Warsaw, Poland; 8 CEA, List, Université Paris-Saclay, Palaiseau, France; 9 Laboratoire LOCEAN/IPSL, Sorbonne Université-CNRS-IRD-MNHN, 75005 Paris, France; 10 Laboratoire d’Excellence “CORAIL,” PSL Research University: EPHE-UPVD-CNRS, USR 3278 CRIOBE, Université de Perpignan, 66100 Perpignan, France; 11 Fondation Tara Océan, Base Tara, 75012 Paris, France; 12 Institut de la Mer de Villefranche Sur Mer, Laboratoire d’Océanographie de Villefranche, Sorbonne Université, 06230 Villefranche-sur-Mer, France; 13 School of Marine Sciences, University of Maine, Orono, ME USA; 14 Shimoda Marine Research Center, University of Tsukuba, Shimoda, Shizuoka, Japan; 15 Institut de Biologie de l’Ecole Normale Supérieure (IBENS), Ecole Normale Supérieure, CNRS, INSERM, Université PSL, 75005 Paris, France; 16 CNRS, Station Biologique de Roscoff, AD2M, UMR 7144, ECOMAP, Sorbonne Université, 29680 Roscoff, France; 17 Department of Earth and Planetary Sciences, Weizmann Institute of Science, 76100 Rehovot, Israel; 18 Institute for Research on Cancer and Aging (IRCAN), Nice, France; 19 Department of Medical Genetics, CHU, Nice, France; 20 CNRS, Laboratoire d’Ecogéochimie des Environnements Benthiques (LECOB), Observatoire Océanologique de Banyuls, Sorbonne Université, 66650 Banyuls sur Mer, France; 21 European Molecular Biology Laboratory, European Bioinformatics Institute (EMBL-EBI), Wellcome Trust Genome Campus, Hinxton, Cambridge, CB10 1SD UK; 22 Department of Biology, Institute of Microbiology and Swiss Institute of Bioinformatics, Vladimir-Prelog-Weg 4, 8093 Zurich, Switzerland; 23 School of Biological and Chemical Sciences, Ryan Institute, University of Galway, University Road, H91 TK33 Galway, Ireland; 24 Department of Microbiology, Oregon State University, 220 Nash Hall, Corvallis, OR 97331 USA; 25 Department of Biology, University of Konstanz, 78457 Konstanz, Germany; 26 Génomique Métabolique, Genoscope, Institut François Jacob, CEA, CNRS, Univ Evry, Université Paris-Saclay, 91057 Evry, France

**Keywords:** Climate sciences, Environmental sciences, Ocean sciences

## Abstract

With climate projections questioning the future survival of stony corals and their dominance as tropical reef builders, it is critical to understand the adaptive capacity of corals to ongoing climate change. Biological mediation of the carbonate chemistry of the coral calcifying fluid is a fundamental component for assessing the response of corals to global threats. The *Tara* Pacific expedition (2016–2018) provided an opportunity to investigate calcification patterns in extant corals throughout the Pacific Ocean. Cores from colonies of the massive *Porites* and *Diploastrea* genera were collected from different environments to assess calcification parameters of long-lived reef-building corals. At the basin scale of the Pacific Ocean, we show that both genera systematically up-regulate their calcifying fluid pH and dissolved inorganic carbon to achieve efficient skeletal precipitation. However, while *Porites* corals increase the aragonite saturation state of the calcifying fluid (Ω_cf_) at higher temperatures to enhance their calcification capacity, *Diploastrea* show a steady homeostatic Ω_cf_ across the Pacific temperature gradient. Thus, the extent to which *Diploastrea* responds to ocean warming and/or acidification is unclear, and it deserves further attention whether this is beneficial or detrimental to future survival of this coral genus.

## Introduction

Ocean warming and acidification threaten the health and survival of tropical coral reefs^1–3^. Projections based on possible future climate scenarios range from a significant decline to the complete disappearance of coral reefs by 2100 (IPCC Special Report, 2018—“Global Warming of 1.5 °C; IPCC Special Report, 2019—Ocean and Cryosphere in a Changing Climate”). For more than a century, increasing emissions of anthropogenic CO_2_^4^ and other greenhouse gases have caused the temperature of the shallow ocean to rise by 0.3–0.6 °C and the pH to fall by ~ 0.1 units (i.e., ocean acidification, OA)^5^. At the same time, the carbonate ion concentration (CO_3_^2−^) and the aragonite saturation state (Ω) in the surface ocean have decreased by ~ 16%^6,7^. Depending on the specific CO_2_ emission scenario^8^, models predict a rise in temperature of several degrees and a further decline in seawater pH (pH_sw_) of 0.14–0.43 by 2100^6,9^. All this could have severe implications for the formation of aragonite in stony corals, including a decline in the calcification rate and skeletal density^9–12^. Several studies have shown that scleractinian (aragonite) corals have an adaptive capacity to maintain calcification under unfavorable environmental conditions^13–15^. They precipitate their calcium carbonate in a biologically controlled manner within a semi-isolated space, known as the extracellular calcifying fluid (cf), located between the skeleton and the calicoblastic epithelium^16^. Corals have developed biological mechanisms to actively concentrate dissolved inorganic carbon (DIC) into the cf and remove protons (i.e., increase the pH_cf_ relative to the ambient seawater). This shifts the DIC equilibrium in favor of [CO_3_^2−^], thus enabling the coral to achieve higher Ω_cf_ values. In particular, by upregulating their cf carbonate chemistry, corals achieve aragonite saturation state levels 4 to 6 times higher than that of seawater^15,17,18^, which promotes the precipitation of CaCO_3_. Moreover, recent intra-colony studies of the genus *Porites* suggested that cf carbonate chemistry varies seasonally, with such variations being regulated by a combination of environmental drivers (e.g., light, temperature, nutrients) and metabolic processes (e.g., metabolic carbon from symbiotic photosynthesis)^19–23^.

**Figure 1:**
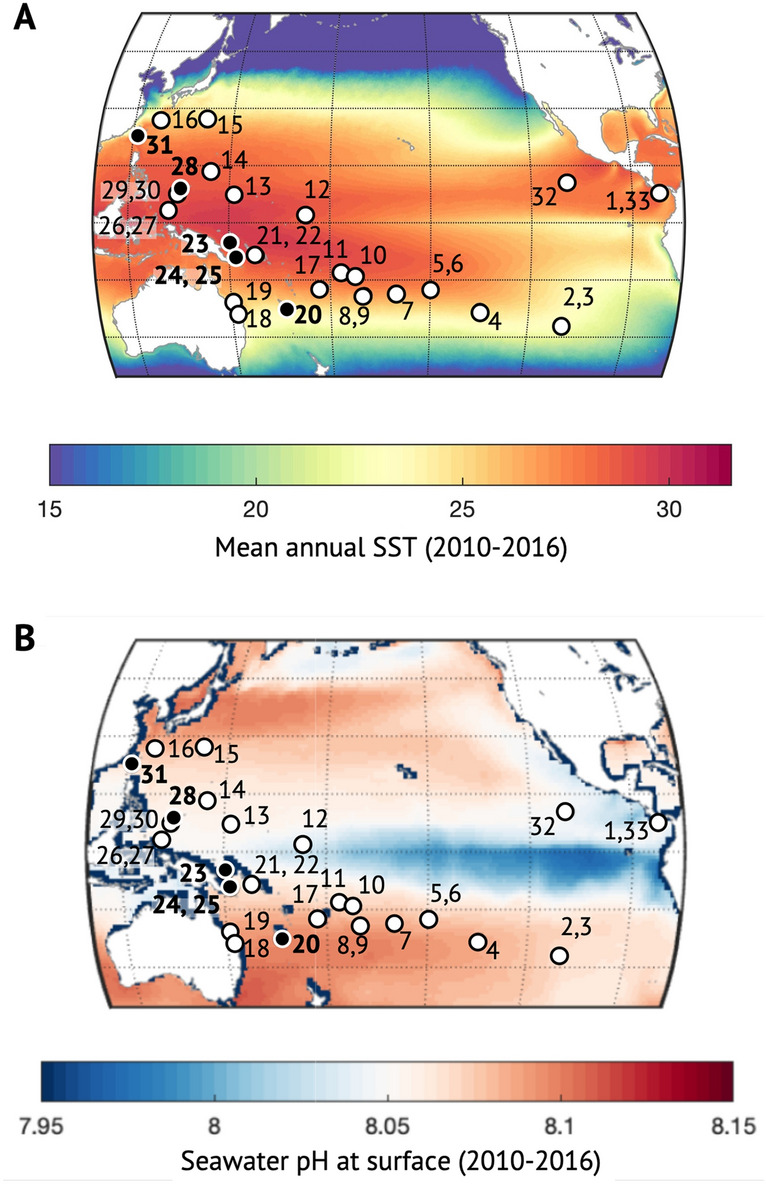
Map of the Pacific Ocean showing the sampling locations of the 39 coral colonies of *Porites* (n = 33) and *Diploastrea* (n = 6), cored during the *Tara* Pacific expedition (2016–2018) used to investigate the chemical properties of the calcification fluid. The 27 white dots correspond to sites where only *Porites* were collected and the 6 black dots correspond to sites where both *Porites* and *Diploastrea* were collected. Numbering, geographical locations, and coral cores are detailed in Table S1. (**A**) Mean sea surface temperature (SST) along the Pacific Ocean for the period 2010–2016. Global annual SSTs were extracted from the MODIS-Aqua satellite and global mapped climatologies established for the period from 2002 to 2018 (NASA Goddard Space Flight Center) (**B**) Mean seawater pH for the period 2010–2016. We used monthly global reconstructed surface ocean *p*CO_2_, air-sea fluxes of CO_2_ and pH to calculate seawater pH and associated uncertainties on a 1° × 1° regular grid^28^. These maps were obtained from an ensemble-based forward feed neural network approach mapping in situ data for surface ocean fugacity (SOCAT data base^29^, https://www.socat.info/) and sea surface salinity, temperature, sea surface height, chlorophyll a, mixed layer depth, and atmospheric CO_2_ mole fraction.

We investigated the carbonate chemistry of the calcifying fluid of two massive and long-lived coral genera (*Porites* and *Diploastrea*) to identify differences and similarities between taxa under identical climatic and hydrological conditions. These coral genera, prevalent reef builders of the Pacific Ocean^24^, have been targeted because of their wide latitudinal distribution, longevity (on the order of centuries), and great potential as palaeoceanographic archives. While *Porites* is known to be among the most resilient corals^25–27^, less is known about the genus *Diploastrea* regarding its stress tolerance. In this study, we compared the calcification and carbonate chemistry up-regulation of *Diploastrea heliopora* and *Porites* corals from across a range of environments. To this, we analyzed the skeletal geochemistry and growth parameters of 39 colonies of *Porites* (n = 33) and *Diploastrea* (n = 6) collected across the tropical Pacific Ocean during the *Tara* Pacific expedition (2016–2018). The collected corals represent a suite of cores exposed to various hydrological conditions of seawater temperature (SST: 22.4–29.8 °C), salinity (SSS: 31.5–36.1), and carbonate chemistry (total scale pH_sw_: 8.01–8.09) (Fig. 1, Table S1, S2). The average chemical composition of the calcifying fluid (pH_cf_, [CO_3_^2−^]_cf_, DIC_cf_, Ω_cf_) was derived from paired boron isotope (δ^11^B) and B/Ca analyses of core-top samples corresponding to the last 6 years of growth (2010–2016; Methods). Based on these data, we assessed the impact of the ambient seawater properties (SST, salinity, carbonate chemistry) on the cf composition of these slow-growing reef-building genera at the Pacific basin scale.

## Results and discussion

**Figure 2:**
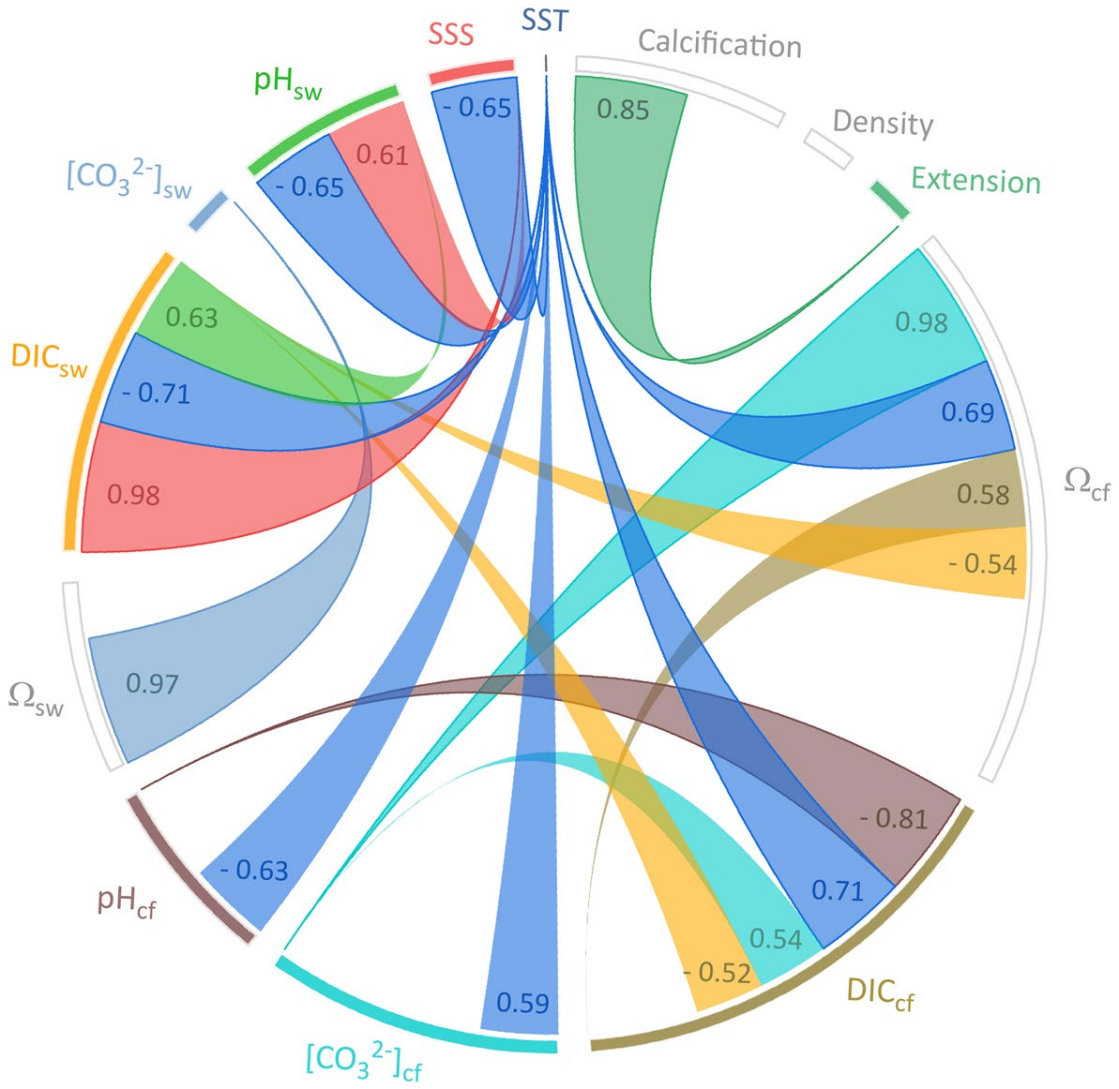
Chord diagram showing the relevant relationships between seawater, calcifying fluid (cf) chemistry, and coral growth parameters for *Porites* across the Pacific Ocean. Each variable is displayed as a node, with the size of the arc corresponding to the strength of the correlation (correlation coefficients are also reported close to each node and in Fig. S2). Links between two nodes displaying a correlation coefficient < 0.5 (for positive correlation) and <|− 0.5| (for negative correlation) are not shown to keep the graph readable and not overwhelming. SST, Sea Surface Temperature; SSS, Sea Surface Salinity; pH_sw_, seawater pH (total scale); [CO_3_^2−^]_sw_, seawater carbonate ion concentration; DIC_sw_, seawater dissolved inorganic carbon; Ω_sw_, aragonite saturation state in seawater; pH_cf_, calcifying fluid pH (total scale); [CO_3_^2−^]_cf_, calcifying fluid carbonate ion concentration; DIC_cf_, calcifying fluid dissolved inorganic carbon; Ω_cf_, aragonite saturation state in the calcifying fluid.

**Figure 3:**
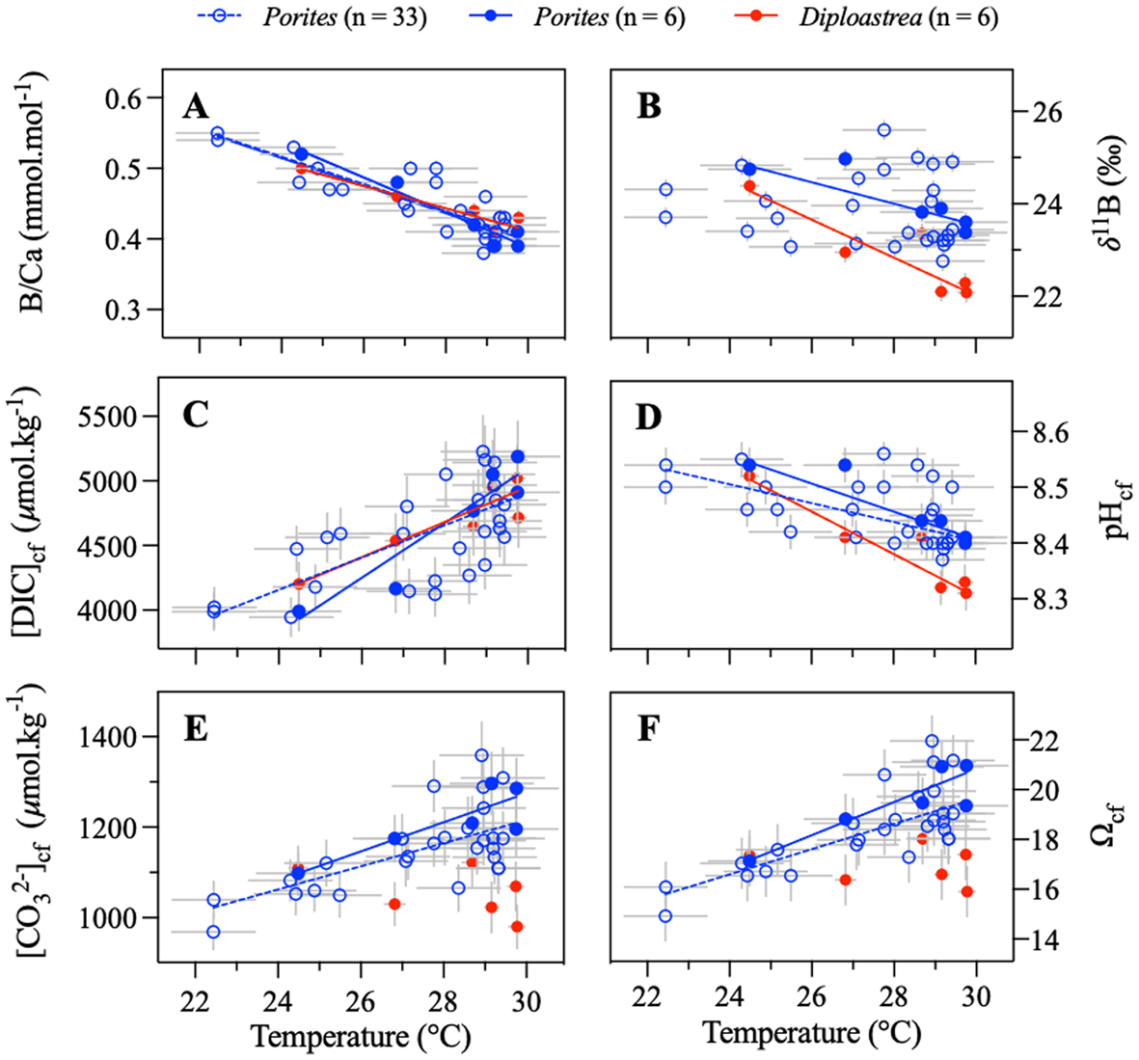
Coral skeletal isotopic composition. (**A**) B/Ca and (**B**) δ^11^B values of *Porites* (n = 33, blue) and *Diploastrea* (n = 6, red) corals across the Pacific Ocean plotted against SST. (**C**–**F**) Carbonate chemistry variables of calcifying fluid calculated for each colony studied here (DIC_cf_, pH_cf_, [CO_3_^2−^]_cf_, Ω_cf_, respectively) plotted against SST. The filled blue and red dots represent the 6 sites where both *Porites* and *Diploastrea* were sampled. Accordingly, the solid blue and red lines correspond to the regression lines for these 6 sites, while the dashed blue lines correspond to the regression lines for all sites where *Porites* were sampled (n = 33). SST values were obtained from the AVHRR-OISSTv2 (0.25 × 0.25°) dataset and correspond to mean values calculated for the period 2010–2016. X and Y errors correspond to 2σ standard deviations of mean SST and 2σ standard errors of measurements or calculations, respectively. Statistical parameters are reported in Table S4.

Coral samples were collected from 33 sites in the Pacific Ocean characterized by different environmental conditions. The mean SST values (integrated over the period 2010–2016) varied between 22.44 °C in Easter Island and 29.76 °C in Papua New Guinea (> 7 °C difference). Mean pH exhibited a relatively small difference between 8.01 in Kiribati and 8.09 in Heron Island (ΔpH = 0.08). Thus, the calculated seawater saturation states (Ω_SW_) varied from 3.21 in Coiba to 3.95 in Moorea (“integrated seawater properties” in Table S2, Fig. S1). Boron-derived values of the cf carbonate chemistry revealed significant differences in [CO_3_^2−^]_cf_ and Ω_cf_ (*P* < 0.05) between *Porites* and *Diploastrea,* with the latter showing lower values (Table S1). Cores of the two genera also showed significantly different linear extension and calcification rates (*P* < 0.05). The comparison between environmental data, growth parameters, and boron-derived cf estimates for *Porites* (Figs. 2, S2) indicates that average pH_cf_ was not controlled by spatial differences in seawater pH or aragonite saturation state (*P* > 0.05). Instead, our data suggest that spatially average pH_cf_ is linked to SST (R = − 0.63, *P* < 0.001) and DIC_sw_ (R = 0.41, *P* = 0.017). While DIC_sw_ showed a significant correlation with salinity (R = 0.98, *P* < 0.001), pH_cf_ was also related to salinity but to a lesser degree (R = 0.35, *P* = 0.046). Similarly, DIC_cf_ was related to SST (R = 0.71, *P* < 0.001). Thus, on spatial scales a strong negative correlation exists between pH_cf_ and DIC_cf_ (R = − 0.81, *P* < 0.001), consistent with other studies at a seasonal scale^20,30,31^. Our results suggest that seawater temperature explains most of the variance in pH_cf_ and DIC_cf_ in *Porites* colonies at a basin-scale (Fig. 2). Similarly, overall observations apply to *Diploastrea* samples, since B/Ca, δ^11^B, DIC_cf_, and pH_cf_ were significantly correlated with seawater temperature (Fig. 3A–D). However, this contrasts with other studies that have shown that seawater pH is the main driver of pH_cf_ on annual and longer time scales, while temperature only plays a secondary role^32,33^. This suggests that the magnitude of SST variations (seasonal vs. annual and temporal vs. spatial) is what effectively controls the relationship between temperature and cf carbonate chemistry. At large, as expected and previously observed in various Indo-Pacific regions^20,30–34^, *Porites* calcification was positively correlated with SST (R = 0.37, *P* = 0.034) and displayed a positive correlation with DIC_cf_ (R = 0.35, *P* = 0.044).

In agreement with recent studies focused on *Porites* at a seasonal scale (Fig. S3;^20,22^), our bulk 6 yr-integrated results show a strong negative relationship between pH_cf_ and SST as well as a positive correlation between DIC_cf_ and SST for both coral genera (Fig. 3). These opposing relationships suggest that corals up-regulate their internal pH in response to temperature-related changes in metabolic DIC, as already posited in previous studies (e.g., by means of higher metabolic DIC availability from algal symbiont photosynthesis at warmer temperatures and/or light)^23,32^.

In this study, for *Porites* we determined a DIC_cf_ increase of 128 ± 23 μmol kg^−1^ per °C, while pH_cf_ decreased by 0.015 ± 0.004 per °C (Fig. 3, Table S4), resulting in an increase in [CO_3_^2^]_cf_ of 29 ± 5 μmol kg^−1^ per °C and higher Ω_cf_ values (~ 21 vs. ~ 16, Fig. 3). The decrease in pH_cf_ with temperature observed at a spatial scale is around three times lower than previous estimates observed at a seasonal scale^32^, and therefore steadier (homeostatic). Besides the notion that temperature influences pH_cf_ up-regulation, our study demonstrates the pH_cf_ up-regulation capacity of *Porites* across stable and warm regions as well as in regions with a large seasonal temperature amplitude and low mean annual temperatures (or mean annual light) (i.e., sub-equatorial vs. equatorial regions). Thus, pH_cf_ up-regulation overcomes the decrease in DIC_cf_ due to colder SSTs (Fig. 3) in sub-equatorial areas to enable coral calcification. Seasonally-resolved *Porites* records of δ^11^B and B/Ca have shown that DIC_cf_ is lower during winter months (i.e., colder temperatures) due to lower metabolic supply of DIC within the calcifying fluid^20^. The supply of this metabolically derived carbon is driven by light and temperature through the respiration of algal symbiont photosynthates^35^, as colder temperatures reduce zooxanthellae activity and reduce the concentration of metabolic DIC in the calcifying fluid. However, higher nutrient availability at higher latitudes may contribute to partially offsetting the detrimental effects caused by the lower metabolic supply of DIC_cf_. The negative correlation between pH_cf_ and DIC_cf_ at a spatial scale in our study is consistent with intracolonial seasonal variations reported in previous studies^20,33^.

The up-regulation of pH_cf_ is one way for corals to compensate for the reduced metabolic carbon input from the algal symbiont and to maintain supersaturated conditions in a biologically controlled compartment with respect to aragonite (Ω_cf_ ~ 5 × Ω_SW_)^20^. This explains why *Porites* corals living in equatorial and sub-equatorial regions display similar Ω_cf_ values, despite their different internal pH_cf_, driven by temperature-dependent DIC_cf_ regulation. Since the photosynthetic activity of the coral associated algal symbiont is presumably reduced at higher latitudes (~ 27° N/S in this study) due to lower light availability compared to equatorial latitudes^36^, corals may use their energy to regulate their cf chemistry, in particular their pH_cf_, to maintain active growth and skeletal accretion.

The results of our study, based on a multi-year sampling strategy of coral core-tops across the Pacific Ocean, are consistent with the calcification model proposed by Ross et al.^30,31^ (Fig. S4), based on a seasonal timescale. The primary mechanism for the up-regulation of pH_cf_ involves the Ca^2+^-ATPase pump, which exchanges one calcium ion for two protons across the cell membrane^37–40^. The removal of H^+^ from the cf increases the diffusion of metabolic CO_2_^38^, which is either protonated to bicarbonate (HCO_3_^−^) by carbonic anhydrase (CA) and/or transported in the form of HCO_3_^−^ by bicarbonate anion transporters (BATs, i.e., through active transport)^41^. Up-regulation of pH_cf_ shifts the DIC equilibrium in favor of CO_3_^2−^, thereby increasing the internal aragonite saturation state to promote skeletal formation^20,38,42,43^.

Our study provides evidence that to maintain growth *Porites* corals up-regulate their pH_cf_ and increase their DIC_cf_ concentration in response to changes in SST across the Pacific Ocean. This physiological mechanism has already been observed for *Porites* on a seasonal timescale in the Great Barrier Reef^20^ (Fig. S3) and Galapagos^22^, as well as during the 1998 bleaching event and associated thermal stress^19^. Here, for the first time we demonstrate that this mechanism applies across a wide range of latitudes and longitudes. The ability of corals to modulate their calcifying fluid chemistry explains their sustained calcification rates, which are primarily driven by temperature and DIC_cf_. *Porites* corals in warmer environments display lower pH_cf_ but higher DIC_cf_ and [CO_3_^2−^]_cf_ (Fig. 3), leading to significantly higher Ω_cf_ compared to the surrounding seawater (Fig. 3) and increasing calcification rates. In contrast to *Porites*, branching corals^31,32^ exhibit higher calcification rates at lower temperatures and higher pH_cf_ and [CO_3_^2−^]_cf_. It is now recognized that the internal modulation of coral calcifying fluid is genus-specific (if not species-specific)^21,31,44^. Our study demonstrates that *Porites* colonies living across a wide range of environments across the Pacific Ocean can modulate their cf chemistry in response to prevalent regional temperature regimes to maintain calcification rates, as previously suggested^20,22^.

**Figure 4:**
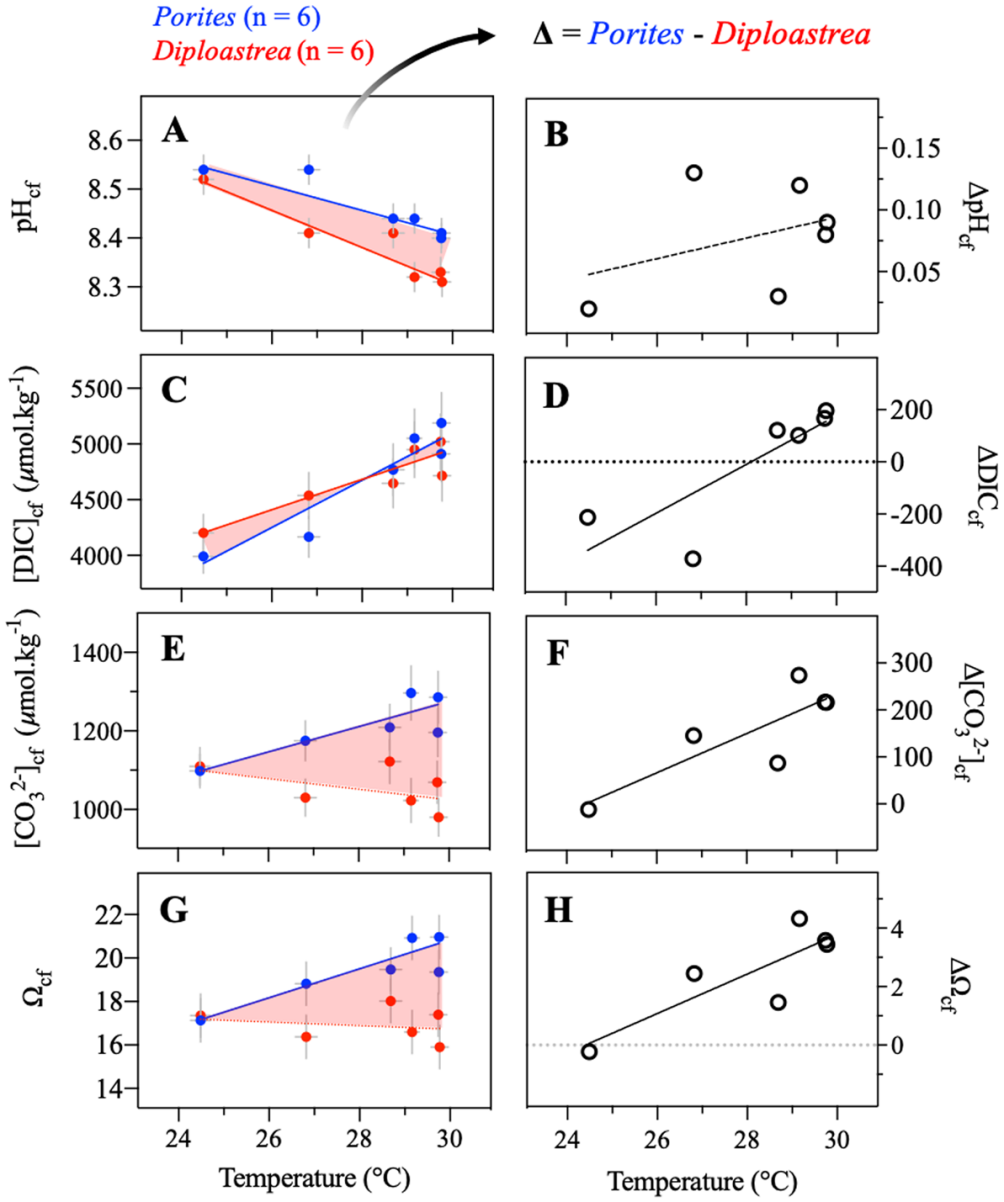
Correlations between SST and calcifying fluid composition in co-occurring *Porites* (n = 6, blue dots) and *Diploastrea* (n = 6, red dots) specimens across the Pacific Ocean. Solid blue and red lines in the left panels indicate significant correlations (*P* < 0.05) for *Porites* and *Diploastrea*, respectively. The dashed lines are not significant at the 95% level. (**A**) pH_cf_, (**C**) DIC_cf_, (**E**) [CO_3_^2−^]_cf_, and (**G**) Ω_cf_. Differences between the two genera are reported in the right panels as Δ (i.e. Δ = *Porites* values – *Diploastrea* values). Black solid lines indicate significant correlations (R^2^ = 0.69–0.82; *P* < 0.05). (**B**) ΔpH_cf_, (**D**) ΔDIC_cf_, (**F**), Δ[CO_3_^2−^]_cf_, and (**H**) ΔΩ_cf_. SST values are from AVHRR-OISSTv2 (0.25 × 0.25°) and correspond to annual integrated 6-yr values during the period 2010–2016. X and Y errors correspond to 2σ standard deviations of mean SST and 2σ standard errors of measurements or calculations, respectively. Statistical parameters are reported in Table S4.

Conversely to *Porites*, the capacity of the long-lived massive coral *Diploastrea* to regulate its internal pH_cf_ had not been studied yet. While *Diploastrea* and *Porites* showed similar decreases in B/Ca ratios with increasing temperature, *Diploastrea* consistently exhibited lower δ^11^B values (and therefore pH_cf_) at the same temperature (Fig. 3), indicating taxa-specific differences with regard to internal pH_cf_ regulation (Fig. 4). In both coral genera, pH_cf_ and DIC_cf_ were positively correlated with the Pacific Ocean temperature. However, at higher temperatures, *Diploastrea* showed a reduced pH_cf_ up-regulation due to a pH_cf_ decrease of − 0.036 ± 0.006 per °C (n = 6), resulting in lower [CO_3_^2−^]_cf_ and Ω_cf_ values (Table S1). This newly discovered finding suggests different mechanisms of calcification control in *Porites* and *Diploastrea*. Our interpretation is that either the *Diploastrea* Ca^2+^-ATPase pump is less effective than that of *Porites* in removing H^+^ from the calcifying cell, or that *Diploastrea* has a mechanism for conserving energy by maintaining stable levels of [CO_3_^2−^]_cf_ and Ω_cf_ (~ 16–18), particularly in regions of higher temperatures (Fig. 3, Table S1).

Response differences of corals to fluctuating temperature (e.g., based on a regional gradient, seasonality, thermal stress) in relation to their calcifying mechanisms have already been documented. It is worth noting that the observed pH_cf_ decrease with SST for *Diploastrea* (− 0.036 per °C, Table S4) is comparable to the mean drop (− 0.03 per °C) recorded for seven symbiotic coral species (4 genera) studied on a seasonal scale in Western Australia over a wide range of latitudes (~ 11°)^31^. However, although the magnitude of change is equivalent, the underlying mechanisms are different. In particular, since DIC_cf_ up-regulation was lower in the Australian corals, the resulting Ω_cf_ values were lower (~ 10–12), and the Ω_cf_ change with temperature varied among species. A notable difference has also been observed between aquaria-reared colonies of *Pocillopora damicornis* and *Stylophora pistillata* grown under various temperature and pCO_2_ conditions^44^ that indicate that only *Pocillopora damicornis* lose its compensatory ability under thermal stress (31 °C vs. 28 °C) with Ω_cf_ values clearly below 10 for different pH_sw_ conditions. Further, during a local thermal stress and bleaching event^45^, the branching coral *Acropora aspera* continued to up-regulate pH_cf_ at high temperatures, while DIC_cf_ up-regulation was significantly impaired, which is in contrast to the response of massive corals examined here. A species-specific response of pH_cf_ and DIC_cf_ up-regulation relative to seawater carbonate chemistry variation and ocean acidification has already been described at a seasonal timescale, showing marked differences in calcification control between massive corals such as *Porites*, *Acropora*, *Psammocora*, and *Pocillopora*^46,47^.

A summary of the taxon-specific responses of cf carbonate chemistry to temperature for the massive corals here studied as Δ (i.e., *Porites* values—*Diploastrea* values) is provided in Fig. 4B,D,F,H. It is apparent that an increase in temperature leads to a substantial increase in Δ, especially for the key parameters [CO_3_^2−^]_cf_ and Ω_cf_ that are directly linked to coral calcification. Thus, despite the elevation of DIC_cf_ at high temperatures, the capacity of *Diploastrea* to increase Ω_cf_ under warmer conditions is clearly different from *Porites*. However, based on our data, *Diploastrea* maintain their capacity to regulate Ω_cf_ and exhibit homeostatic control of the aragonite saturation state independently of geographic location or temperature. This indicates that the pronounced drop of pH_cf_ up-regulation with temperature (− 0.036 ± 0.006 per °C, n = 6) is sufficiently compensated by the buffering capacity and DIC_cf_ increase (129 ± 30 per °C, n = 6). Therefore, the calcification ability of *Diploastrea* is less sensitive to ocean temperature changes compared to *Porites* when we consider the key parameters [CO_3_^2−^]_cf_ and Ω_cf_. This may suggest that calcification rate for *Diploastrea* is potentially less variable in space and time (seasonal amplitudes) compared to *Porites*. The mechanism observed in *Diploastrea* appears to resemble the one described by Georgiou et al. (2015)^46^, which demonstrated the capacity to maintain calcification irrespective of environmental differences (i.e., homeostasis), suggesting a similar mechanism in *Diploastrea*. By stabilizing its chemical composition, even at high temperatures, *Diploastrea* can achieve optimal calcification. Further studies are required to evaluate such a hypothesis but also better understand the potential impact of the lower Ca^2+^-ATPase pump efficiency and pH_cf_ up-regulation posited for *Diploastrea* at higher temperatures on calcification, especially in the context of climate change. It should also be noted that Ω_cf_ values (~ 16–18) were calculated assuming Ca^2+^ concentrations in the calcifying fluid similar to that of the seawater. However, this assumption requires further investigation for *Diploastrea* and *Porites*, as recent studies have shown substantial variations in Ca^2+^ concentration of the calcifying fluid^15^.

Our study across the Pacific Ocean confirms the ability of the massive reef-building *Porites* genus to modulate the composition of its calcifying fluid in response to seawater temperature and carbonate chemistry, as observed for other scleractinian corals at different locations or exposed to disparate environmental conditions. For *Porites*, an upward shift in pH_cf_ and DIC_cf_ (relative to seawater) driven by temperature changes is the presumed mechanism for *Porites* to compensate for the impact of future thermal stress events or ocean warming on calcification in the Pacific Ocean. Further, our study demonstrates that SST rather than pH_sw_ or Ω_sw_, is the key parameter controlling *Porites* calcifying fluid properties, through the activity of the zooxanthellae. Thus, *Porites* is able to adapt its metabolism to increases in seawater temperature, heralding the adaptive potential of *Porites* to maintain or reinforce a high aragonite saturation state Ω_cf_ and calcification capacity in the face of climate change. Importantly, however, our results do not rule out that ocean acidification^12,33,46–50^ or other environmental factors, including changes in light conditions^23^, may affect coral calcification locally in the near future. Our study also demonstrates biological control of the calcification process is taxon-specific. We show that *Diploastrea* displays a different strategy than *Porites* at high temperatures (28–30 °C), in that it maintains consistent calcification rates irrespective of the prevailing environment. Species-specific differences need to be thus considered when forecasting coral future survival^51^. Further investigations of the response of *Diploastrea* corals at annual and seasonal timescales will reduce uncertainties and better constrain the range of their homeostatic ability to calcify at warming water temperatures. A part of these future research works will be conducted in the new program COR-Resilience (2023–2028) recently funded by the French National Research Agency.

## Methods

### Coral core sampling

Thirty-nine coral cores (40–150 cm long) were collected from living *Porites* and *Diploastrea* colonies during the *Tara* Pacific expedition^52^ between 2016 and 2018 using a hydraulic drill (Stanley®) with a 7 cm diameter corer. After drilling, a cement plug was placed in the opening to facilitate the recovery of the colonies. Details of sampling locations, depth, date and hydrological conditions are reported in Table S1. Cores from both genera were collected at six locations (New Caledonia, three in Papua New Guinea, Palau, and Taiwan; black dots in Fig. 1), allowing comparison of results and evaluating genus effects in 6 different hydrological environments.

### Coral growth parameters

Skeletal density (g cm^−3^) was measured at DOSEO Platform (CEA) in Paris-Saclay using a Discovery CT750 high-definition Computed Tomography X-ray system operated at 120 kV^53,54^. The spatial × resolution of the scans along the maximum growth axis of the colonies was 0.625 mm, while y (width) and z (height) resolutions ranged from about 0.59–0.79 mm. For each coral core, a mean density was calculated by averaging density measurements along 3 parallel transects corresponding to the growth period 2010–2016 (Fig. S5). Analytical precision of the CT density values was estimated to 4% (2σ) based on repeated measurements (n = 10) of 3 coral standards and uncertainties of the calibration curve^53,54^. CT scans were also used to quantify the linear extension rate (mm yr^−1^) based on the density banding pattern. Mean linear extension rates (upward linear growth) were determined for each coral core by measuring the distance between successive low-density bands over the last 6 years of growth (2010–2016), excluding the tissue layer (Fig. S5). The uncertainty of the linear extension rates was calculated from two sets of measurements (Table S3) performed with CT-scans and was 4% (2σ). Finally, coral calcification rate (g cm^−2^ yr^−1^) was calculated as the product of the annual linear extension rate (cm yr^−1^) and the skeletal density (g cm^−3^) with an overall uncertainty of 6% (2σ).

### Coral powder sampling

Core-top samples for geochemical analyses (~ 1000 mg) were collected using a dental drill (Dremel®) with a 0.17 mm thick diamond-encrusted blade along transects (Fig. S5A) parallel to the maximum growth axis corresponding to the last 6 years of growth (2010–2016), excluding the tissue layer, and according to the density banding pattern observed on CT-scans (Fig. S5B). Each aragonite piece corresponding to the period 2010–2016 was finely crushed and thoroughly homogenized in an agate mortar. Prior to elemental (B/Ca) and isotopic (δ^11^B) analysis, 100 mg of powder was sub-sampled and cleaned from organic contaminations following an oxidative cleaning protocol^55^. Finally, they were dissolved in 3 mL of 4 wt% HNO3 for B/Ca and δ^11^B analysis. We opted here for a bulk sampling strategy (i.e. a single large sampling integrating 6 years of growth including all skeletal structures, 2–5 cm in length; 1–2 cm in width/thickness, Fig. S5) to avoid potential geochemical biases associated with the coral mesostructures/microstructures^56^ and strong seasonal δ^11^B and B/Ca variability^57^. The integration of several years into a single sample analysis, was intended to ensure a constant mixing ratio of coral micro- and mesostructures (COCs, aragonite fibers, thecal wall, and columella) with minor effects on boron geochemistry (see below δ^11^B section), especially for *Diploastrea*^58^. Among the 39 samples analysed in the present study, the uncertainty due to the possible inclusion of skeleton from other years in the coral samples was estimated for carbonate chemistry and SST and was considered negligible^59^. For example, uncertainties in SST due to our multiple-year sampling strategy would be less than 0.2 °C.

### B/Ca ratio

A 15 μL aliquot of dissolved powder was diluted in ~ 2 mL of 0.5N HNO3 to obtain a 100 ppm Ca solution for B/Ca analysis. ^11^B and^43,44^ Ca isotopes were measured using a Thermo Scientific ICP-MS Xseries^II^ at the Laboratoire des Sciences du Climat et de l’Environnement (LSCE) in Gif-sur-Yvette (France) following the LSCE’s analytical revised protocol^55^. The external reproducibility of the B/Ca ratio was determined based on multiple measurements of two standard solutions (JCp-1 and a new home-made coral standard M1P-p). The reproducibility was better than 2% (2σ RSD) and the B/Ca value for JCp-1 (459 ± 9 µmol mol^−1^; n = 7) agrees with the robust average value reported in Hathorne et al. (2013)^60^ for this interlaboratory standard. The long-term B/Ca value for M1P-p was 494 ± 3 µmol mol^−1^ (n = 25).

### Boron isotopes analysis (δ^11^B)

Boron was purified using a batch protocol^61–63^ and its isotopes (^11^B and ^10^B) were measured using a Thermo Scientific Multi-Collector ICP-MS Neptune^Plus^ hosted at LSCE. 100–200 ppb boron solutions were introduced into the mass spectrometer through a PFA-50 μL min^−1^ nebulizer and a micro-cyclonic chamber. Instrumental mass fractionation and long-term drift of the ^11^B/^10^B ratio were systematically corrected by applying a standard-sample-standard bracketing protocol, and using a M1P-p solution with a typical measured δ^11^B value of 25.20 ± 0.25‰ (2σ, n = 50). Further details in Wu et al.^63^. Under this condition, the mean δ^11^B value obtained for *Porites* JCp-1 is 24.28 ± 0.36‰ (2σ, n = 15), which agrees well with the robust mean reported in Gutjahr et al.^64^ (24.25 ± 0.22‰). Each sample was measured three times from the same solution and the precision (2σ) was in general better than 0.3‰. In addition, to evaluate possible effects of our sub-sampling strategy of each core-top powder and its heterogeneity, we also analysed eleven 100 mg-sub-samples of a *Diploastrea* homogenised powder from the core I28S3-*D* from Taiwan (sample I28S3D-OM) and six of *Porites* from the core I23S2-*P* from Papua New Guinea (sample I23S2*P*-38). The reproducibility obtained for δ^11^B measurements was ± 0.36‰ (2σ, n = 11) for *Diploastrea* and ± 0.18‰ (2σ, n = 6) for *Porites*, respectively. Even though the value for *Diploastrea* is twice that of *Porites*, possibly due to the effects of genus-specific micro- and mesostructures^58^, the uncertainties remain indistinguishable from the analytical uncertainties determined for the *Porites* standards (± 0.3‰), and significantly smaller than the difference observed between the two genera for each site, which ranges from 0.4 to 2‰. We also tested possible effects of genus-specific micro- and mesostructures (i.e. septa or columella) on the *Diploastrea* skeleton by taking 2 samples from the same core-top portion of the I21S2c17 colony (over the period 2010–2016; e.g. Fig. S5). The results show no major effect on δ^11^B and B/Ca composition, with the 2 samples having the same values within error (i.e. 24.00 ± 0.30‰ and 24.49 ± 0.30%, respectively; the difference in B/Ca was less than 2%). These results suggest that our sampling strategy that integrates multiple years into a single sample for each site avoids potential geochemical biases related to coral microstructures and different mixing ratios.

### Calcifying fluid carbonate chemistry

The pH of the calcifying fluid (pH_cf_) was calculated from the boron isotopic composition of the coral skeleton (δ^11^B coral) according to the following Eq. ^65,66^:

$${\mathrm{pH}}_{\mathrm{cf}}= {\mathrm{pK}}_{\mathrm{B}}-\mathrm{log }\left[\frac{{\updelta }^{11}{\mathrm{B}}_{\mathrm{sw}}-{\updelta }^{11}{\mathrm{B}}_{\mathrm{coral}}}{{\mathrm{\alpha }}_{\mathrm{B }}{\updelta }^{11}{\mathrm{B}}_{\mathrm{coral}}-{\updelta }^{11}{\mathrm{B}}_{\mathrm{sw}}+1000 ({\mathrm{\alpha }}_{\mathrm{B }}-1 )}\right]$$

where δ^11^B_sw_ is the boron isotopic composition of seawater (39.61‰; Foster et al.^67^) and α_B_ is the isotopic fractionation factor (1.0272)^68^. The dissociation constant of boric acid (pK_B_) in seawater^69^ was calculated from the temperature (i.e. mean OiSST), salinity (i.e. mean EN4) and depth (pressure) for each sampling location.

The carbonate ion concentration in the calcifying fluid was calculated using B/Ca according to the following equation^19^:

$$\left[ {{\text{CO}}_{3}^{2 - } } \right]_{{{\text{cf}}}} = {\text{K}}_{{\text{D}}}^{{{\text{B}}/{\text{Ca}}}} * \left[ {{\text{B}}\left( {{\text{OH}}} \right)_{4}^{ - } } \right]_{{{\text{cf}}}} /\left( {{\text{B}}/{\text{Ca}}} \right)_{{{\text{CaCO}}_{3} }}$$

where [B(OH)_4_]_cf_ is the concentration of borate ion in the calcifying fluid derived from δ^11^B-pH_cf_ and corrected for SST, salinity, and depth. K_D_^B/Ca^ is the distribution coefficient for boron between aragonite and seawater^70^ that has been refit as a function of [H^+^]^57^, and $\left( {{\text{B}}/{\text{Ca}}} \right)_{{{\text{CaCO}}_{3} }}$ is the elemental ratio of boron to calcium measured in the coral skeleton. To estimate ${\left[{\mathrm{B}\left(\mathrm{OH}\right)}_{4}^{-}\right]}_{\mathrm{cf}}$, we assume that the concentration of total boron in the calcifying fluid is only salinity dependent and is equal to that of the surrounding seawater. Dissolved inorganic carbon (DIC_cf_) and aragonite saturation state (Ω_cf_) in the calcifying fluid were estimated from pH_cf_ and [CO_3_^2−^]_cf_, using CO2SYS.m Matlab script^71,72^, with carbonate species dissociation from Dickson and Millero^73^ and Mehrbach et al.^74^, borate and sulfate dissociation from Dickson^69,75^ and aragonite solubility from Mucci et al.^76^.

For Ω_cf_ calculations, it was assumed that [Ca^2+^] values in calcifying fluids (~ 13 mM) are higher than seawater values (~ 10.5 mM), based on the results reported in Sevilgen et al.^77^. These authors measured Ca^2+^ concentration in the cf of the growing edge of *Stylophora pistillata* through direct in vivo measurements (microsensors) and found that this coral elevated [Ca^2+^] by about 2 ± 2 mM compared to seawater values for both light and dark conditions. They also observed substantial Ca^2+^ variations in cf, indicating temporal and spatial variation in Ω_cf_. Elevated Ca^2+^ concentration (+ 25%) in cf was also inferred by DeCarlo et al^78^ for *Pocillopora damicornis* using indirect methods (Raman spectroscopy and boron isotopes). However, as previously tested by Thompson et al.^79^, this [Ca^2+^] upregulation compared to seawater only affects the absolute magnitude of the aragonite saturation state in the cf, not the relative differences between colonies, sites, or time periods. Therefore, we consider that our main findings and conclusions on the aragonite saturation state are here independent of the Ca^2+^ concentration and that further studies would be useful to better quantify the calcium concentration in the calcifying fluids of massive corals. The Ω_cf_ values displayed in Table S1 and discussed in this study were calculated by considering no difference in [Ca^2+^] between calcifying fluid and seawater. These values in massive corals would increase of ~ 4 unit if we consider that Ca^2+^ is around 25% more concentrated in fluids.

Uncertainties in pH_cf_ and [CO_3_^2−^]_cf_ were obtained using the boron systematics package of DeCarlo et al.^78^ and were less than 0.03 pH units and 74 μmol kg^−1^ respectively. The uncertainties of DIC_cf_ and Ω_cf_, calculated using the error m script Matlab^80^, were less than 278 μmol kg^−1^ and 1.06, respectively.

### Environmental data (SST, salinity, and seawater carbonate chemistry)

Key environmental parameters, including SST, salinity, total alkalinity, and dissolved inorganic carbon, were acquired as discrete measurements at coral sites (few meters from the coral drilling sites) during the *Tara *Pacific expedition. Ambient seawater temperature and salinity were obtained using a CTD (± 0.1 °C and ± 0.01, respectively), whereas seawater samples for TA and DIC measurements were collected in 500 mL glass-bottles. The unfiltered seawater samples were poisoned with HgCl_2_ and stored onboard at room temperature prior to TA and DIC analyses performed at the SNAPOCO2 facility at Sorbonne University in Paris, France^81^ following the SNAPOCO2 protocol^82,83^. Raw results were recently described in (Lombard et al., 2023)^84^ and are now available in the PANGEA data base^85^. Calibrated Certified Reference Material (CRM, Dickson et al.^86^) were regularly analyzed (CRM Batches 155, 165, 173 and 182). External reproducibility obtained from repeated measurements of standard solutions was ~ 3 μmol kg^−1^ (0.15%) for both parameters. Total seawater carbonate chemistry (i.e. pH_sw_, [CO_3_^2−^]_sw_, DIC_sw_, and Ω_sw_) was calculated using the CO2SYS.m Matlab script^72^. Similar, to SST and salinity, discrete in situ measurements represent only a snapshot of the carbonate chemistry variability of the coral reef, which is affected by diurnal and seasonal cycles mainly related to temperature-driven *p*CO_2_ solubility and other local factors (e.g. residence time of waters in the reef, balance between production and respiration). To overcome this limitation, we used SST from the AVHRR-OISSTv2 dataset with a spatial resolution of 0.25° × 0.25°^87,88^, salinity from the EN4 dataset at 1° × 1°^89,90^, and pH_sw_ values from the Operational Mercator Ocean biogeochemical global ocean analysis and forecast system at 0.25 × 0.25°, based on in situ DIC and TA measurements from the GLODAPv2 database^91^. Mean SST and salinity values for each site were calculated by averaging monthly data from January 2010 to December 2016 (the period covered by the coral portion collected for the geochemical analyses).

Annual mean TA values were derived from salinity based on the following linear equation: TA (μmol kg^−1^) = 2299 × (salinity/35) for tropical and subtropical regions^92^. Finally, seawater [CO_3_^2−^] and Ω were calculated using CO2SYS.m Matlab script^72^ with the values of SST, salinity, TA, and pH.

All the environmental data are reported in Table S2 and Fig. S1.

### Statistical data treatment

Pearson correlation coefficients were used to assess the degree of correlation between discrete *Tara* seawater measurements and values derived from the different datasets. Outliers were identified using the ROUT test and excluded if present. Independent two-sample t-tests were used to detect significant differences in growth parameters between *Porites* and *Diploastrea* samples. The non-parametric Spearman’s rank-order correlation was performed to determine the strength and direction of the association between two ranked variables illustrated in Figure S1 and S2. Rank correlations sort observations by rank and compute the level of similarity between the rank of the variables. R coefficients are always between − 1 and 1 with values close to the extremity indicating strong relationships. The correlation matrixes (Fig. S2) represent the pair correlation of all the variables (i.e. seawater temperature, salinity and carbonate chemistry, coral cf chemistry, and growth parameters). The significance level of statistical tests is expressed with *p*-values (P) with a threshold of significance defined at 0.05 (5%) or 0.001 (1‰). Statistical data treatment was performed using PRISM software.

## Supplementary Information

Supplementary Information.

## Data Availability

All data generated or analyzed during this study are included in the publication or in the supplementary information files. Data will be also publicly available on the PANGAEA data repository.
