# Supplementary material for: Differences in carbonate chemistry up-regulation of long-lived reef-building corals
Source: Sci Rep. 2023 Jul 18;13:11589. doi: 10.1038/s41598-023-37598-9 (PMC10353996; doi:10.1038/s41598-023-37598-9)
Supplement: Supplementary file 1 — Supplementary Information. [file 41598_2023_37598_MOESM1_ESM.docx]

**Supplementary material**

**DIFFERENCES IN CALCIFICATION OF LONG-LIVED REEF-BUILDING CORALS**

Marine Canesi1,2*, Eric Douville1, Paolo Montagna3,4,, Marco Taviani4,5, Jarosław Stolarski6, Louise Bordier1, Arnaud Dapoigny1, Gninwoyo Eric Hermann Coulibaly1, Anne-Catherine Simon7, Mathieu Agelou7, Jonathan Fin8, Nicolas Metzl8, Guillaume Iwankow9, Denis Allemand2, Serge Planes9, Clémentine Moulin10, Fabien Lombard11, Guillaume Bourdin12, Romain Troublé10, Sylvain Agostini13, Bernard Banaigs9, Emilie Boissin9, Emmanuel Boss12, Chris Bowler14, Colomban de Vargas15, Michel Flores16, Didier Forcioli17, Paola Furla17, Eric Gilson17,18, Pierre E. Galand19, Stéphane Pesant20, Shinichi Sunagawa21, Olivier Thomas22, Rebecca Vega Thurber23, Christian R. Voolstra24, Patrick Wincker25, Didier Zoccola2, Stéphanie Reynaud2

1 Laboratoire des Sciences du Climat et de l’Environnement, LSCE/IPSL, UMR 8212 CEA- CNRS-UVSQ, Université Paris-Saclay, 91191 Gif-sur-Yvette, France

2 Centre Scientifique de Monaco, 8 Quai Antoine Ier, 98000 Monaco, Principality of Monaco, Monaco – LIA ROPSE, Laboratoire International Associé Université Côte d'Azur - Centre Scientifique de Monaco

3 Institute of Polar Sciences (ISP), CNR, Via Gobetti 101, 40129 Bologna, Italy
4 Stazione Zoologica Anton Dohrn, Villa Comunale, 80121, Napoli, Italy
5 Institute of Marine Sciences (ISMAR), CNR, Via Gobetti 101, 40129 Bologna, Italy
6 Institute of Paleobiology, Polish Academy of Sciences, PL-00-818 Warsaw, Poland
7 Université Paris-Saclay, CEA, List, Palaiseau, France
8 Laboratoire LOCEAN/IPSL, Sorbonne Université-CNRS-IRD-MNHN, Paris, 75005, France 9 Laboratoire d’Excellence “CORAIL,” PSL Research University: EPHE-UPVD-CNRS, USR

3278 CRIOBE, Université de Perpignan, 66100 Perpignan, France

10 Fondation Tara Océan, Base Tara, 75012 Paris, France

11 Sorbonne Université, Institut de la Mer de Villefranche sur mer, Laboratoire d’Océanographie de Villefranche, 06230 Villefranche-sur-Mer, France

12 School of Marine Sciences, University of Maine, Orono, Maine, United States of America

13 Shimoda Marine Research Center, University of Tsukuba, Shimoda, Shizuoka, Japan

14 Institut de Biologie de l'Ecole Normale Supérieure (IBENS), Ecole normale supérieure, CNRS, INSERM, Université PSL, 75005 Paris, France

15 Sorbonne Université, CNRS, Station Biologique de Roscoff, AD2M, UMR 7144, ECOMAP, 29680 Roscoff, France

16 Weizmann Institute of Science, Department of Earth and Planetary Sciences, 76100 Rehovot, Israel

17 Université Côte d'Azur, CNRS, INSERM, Institute for Research on Cancer and Aging, Nice (IRCAN) – LIA ROPSE, Laboratoire International Associé Université Côte d'Azur - Centre Scientifique de Monaco – U FR

18 Department of Medical Genetics, CHU Nice, France

19 Sorbonne Université, CNRS, Laboratoire d’Ecogéochimie des Environnements Benthiques (LECOB), Observatoire Océanologique de Banyuls, 66650 Banyuls sur mer, France

20 European Bioinformatics Institute (EMBL-EBI), European Molecular Biology Laboratory, Wellcome Trust Genome Campus, Hinxton, Cambridge CB10 1SD
United Kingdom

21 Department of Biology, Institute of Microbiology and Swiss Institute of Bioinformatics, Vladimir-Prelog-Weg 4, 8093 Zurich, Switzerland

22 Marine Biodiscovery Laboratory, School of Chemistry and Ryan Institute, National University of Ireland, Galway, Ireland

23 Oregon State University, Department of Microbiology, 220 Nash Hall, 97331Corvallis OR, USA

24 Department of Biology, University of Konstanz, 78457 Konstanz, Germany

25 Génomique Métabolique, Genoscope, Institut François Jacob, CEA, CNRS, Univ Evry, Université Paris-Saclay, 91057 Evry, France

* **Corresponding author** at: Laboratoire des Sciences du Climat et de l’Environnement (LSCE), Orme des Merisiers, 91191 Gif-sur-Yvette, France.
*E-mail address*: marine.canesi@gmail.com (Marine Canesi).

**Table S1.** Geochemistry (B/Ca, δ11B), carbonate chemistry (pHcf, [CO32-]cf, [DIC]cf, Ωcf) of the calcifying fluid, and growth parameters (extension rate, density, calcification rate) of 39 massive coral colonies across the Pacific Ocean. *Porites* spp. (n = 33) and *Diploastrea* spp. (n = 6).

|  |  |  |  | |  | |  |  |  |  | **CORAL PROPERTIES** | | | | | | | | |  |
| --- | --- | --- | --- | --- | --- | --- | --- | --- | --- | --- | --- | --- | --- | --- | --- | --- | --- | --- | --- | --- |
|  |  |  |  |  |  |  |  |  |  |  | Skeletal geochemistry | | Calcifying fluid properties | | | | Growth parameters | | |  |
| Fig 1 nb. | Location | Sample | Latitude | | Longitude | | Depth | Sampling date | SST | Salinity | B/Ca | δ^11^B | pH_cf_ | [CO_3_^2-^]_cf_ | DIC_cf_ | Ω_cf_ | Extension | Density | Calcification |  |
|  |  |  |  |  |  |  | (m) |  | °C |  | mmol·mol^-1^ | ‰ |  | 𝜇mol·kg^-1^ | 𝜇mol·kg^-1^ |  | cm·yr^-1^ | g·cm^-3^ | g·cm^-2^·yr^-1^ |  |
|  | ***Porites* spp.** |  |  |  |  |  |  |  |  |  |  |  |  |  |  |  |  |  |  |  |
| 1 | Coiba (Panama) | I2S3c21 | N | 7°12' | W | 81°47' | 8 | 23/07/2016 | 28.59 | 31.52 | 0.43 | 25.00 | 8.54 | 1198 | 4267 | 19.72 | 0.88 | 1.48 | 1.30 |  |
| 2 | Anakena, Isla de Pascua (Chile) | I4S1 | S | 27°04' | W | 109°19' | 14 | 03/09/2016 | 22.45 | 36.04 | 0.54 | 24.31 | 8.54 | 1040 | 4021 | 16.09 | 1.00 | 1.50 | 1.50 |  |
| 3 | Motu Taka Rua, Isla de Pascua (Chile) | I5S4 | S | 27°04' | W | 109°19' | 14 | 07/09/2016 | 22.44 | 36.05 | 0.55 | 23.71 | 8.50 | 969 | 3989 | 14.92 | 1.33 | 1.19 | 1.58 |  |
| 4 | Tekava, Gambier (French Polynesia) | I6S2 | S | 23°09' | W | 134°50' | 9 | 23/09/2016 | 25.17 | 36.08 | 0.47 | 23.69 | 8.46 | 1121 | 4563 | 17.59 | 0.83 | 1.53 | 1.27 |  |
| 5 | Moorea (French Polynesia) | I7S1c1 | S | 17°28' | W | 149°48' | 3 | 08/11/2016 | 27.77 | 35.91 | 0.48 | 25.6 | 8.56 | 1291 | 4224 | 20.6 | 1.20 | 1.32 | 1.58 |  |
| 6 | Moorea (French Polynesia) | I7S3c2 | S | 17°29' | W | 149°45' | 13 | 09/11/2016 | 27.77 | 35.86 | 0.50 | 24.74 | 8.50 | 1164 | 4124 | 18.4 | 1.25 | 1.44 | 1.80 |  |
| 7 | Aitutaki, Cook (New Zeland) | I8S1c3 | S | 18°50' | W | 159°48' | 14 | 14/11/2016 | 27.09 | 35.67 | 0.44 | 23.14 | 8.41 | 1125 | 4801 | 17.79 | 1.00 | 1.24 | 1.24 |  |
| 8 | S Niue (New Zeland) | I9S1c4 | S | 19°06' | W | 169°54' | 12 | 22/11/2016 | 27.00 | 35.44 | 0.45 | 23.96 | 8.46 | 1174 | 4592 | 18.65 | 1.20 | 1.62 | 1.94 |  |
| 9 | W Niue (New Zeland) | I9S2c5 | S | 18°59' | W | 169°54' | 15 | 24/11/2016 | 27.14 | 35.43 | 0.50 | 24.55 | 8.50 | 1135 | 4145 | 17.98 | 0.86 | 1.51 | 1.30 |  |
| 10 | Upolu (Samoa) | I10S0c6 | S | 13°50' | W | 172°04' | 9 | 30/11/2016 | 28.81 | 35.17 | 0.42 | 23.21 | 8.40 | 1154 | 4852 | 18.53 | 1.00 | 1.39 | 1.39 |  |
| 11 | CSR 11, Wallis (France) | I11S1c7 | S | 13°18' | W | 176°12' | 20 | 18/12/2016 | 28.97 | 35.06 | 0.46 | 24.29 | 8.46 | 1171 | 4350 | 18.77 | 0.67 | 1.35 | 0.90 |  |
| 12 | Abaiang (Kiribati) | I13S3c8 | N | 01°43' | E | 172°58' | 7 | 09/01/2017 | 28.97 | 34.89 | 0.40 | 23.29 | 8.40 | 1242 | 5164 | 19.94 | 1.33 | 1.21 | 1.61 |  |
| 13 | Pisinun, Chuuk (Micronesia) | I14S2c9 | N | 07°08' | E | 151°53' | 8 | 22/01/2017 | 29.23 | 34.09 | 0.41 | 23.11 | 8.39 | 1134 | 4851 | 18.40 | 1.00 | 1.39 | 1.39 |  |
| 14 | Guam (USA) | I15S1c10 | N | 13°14' | E | 144°38' | 9 | 29/01/2017 | 28.92 | 34.46 | 0.38 | 24.05 | 8.45 | 1359 | 5226 | 21.96 | 0.75 | 1.61 | 1.21 |  |
| 15 | Ogasawara (Japan) | I16S2c11 | N | 27°05' | E | 142°13' | 5 | 10/02/2017 | 24.30 | 34.78 | 0.53 | 24.83 | 8.55 | 1082 | 3945 | 17.04 | 0.67 | 1.32 | 0.88 |  |
| 16 | Sesoko, Okinawa (Japan) | I17 | N | 26°38' | E | 127°51' | 6 | 13/04/2017 | 24.88 | 34.71 | 0.50 | 24.06 | 8.50 | 1060 | 4180 | 16.72 | 1.14 | 1.09 | 1.24 |  |
| 17 | NW Fiji | I18S1 | S | 17°22' | E | 177°07' | 7 | 03/06/2017 | 28.03 | 35.08 | 0.41 | 23.07 | 8.40 | 1177 | 5051 | 18.79 | 1.25 | 1.27 | 1.59 |  |
| 18 | Heron, S GBR (Australia) | I19S1c13 | S | 23°26' | E | 151°54' | 7 | 30/08/2017 | 24.43 | 35.39 | 0.48 | 23.41 | 8.46 | 1053 | 4473 | 16.55 | 1.00 | 1.13 | 1.13 |  |
| 19 | SW Cockatoo Reef, GBR (Australia) | I19S3c14 | S | 20°46' | E | 150°53' | 7 | 03/09/2017 | 25.49 | 35.36 | 0.47 | 23.07 | 8.42 | 1050 | 4591 | 16.54 | 1.50 | 1.28 | 1.92 |  |
| 20 | Noumea Lagoon (New Caledonia) | I21S2c16 | S | 22°32' | E | 166°26' | 12 | 25/09/2017 | 24.49 | 35.44 | 0.52 | 24.75 | 8.54 | 1098 | 3990 | 17.14 | 1.00 | 1.45 | 1.45 |  |
| 21 | E Vangunu (Salomon) | I22S2c1 | S | 08°36' | E | 158° 11' | 4 | 23/10/2017 | 29.43 | 34.61 | 0.43 | 24.91 | 8.50 | 1309 | 4566 | 21.18 | 1.50 | 1.37 | 2.06 |  |
| 22 | E Vangunu (Salomon) | I22S2c2 | S | 08°36' | E | 158° 11' | 4 | 23/10/2017 | 29.43 | 34.61 | 0.42 | 23.44 | 8.41 | 1175 | 4818 | 19.04 | 1.29 | 1.25 | 1.61 |  |
| 23 | Losuia, Tabungora Island (PNG) | I23S2 | S | 9°21' | E | 152°02' | 13 | 06/11/2017 | 28.69 | 34.63 | 0.42 | 23.82 | 8.44 | 1209 | 4769 | 19.48 | 1.00 | 1.54 | 1.54 |  |
| 24 | Kimbe, Hoskins District (PNG) | I24S2c2 | S | 5°05' | E | 150°12' | 17 | 17/11/2017 | 29.75 | 34.41 | 0.41 | 23.38 | 8.40 | 1196 | 4911 | 19.35 | 1.17 | 1.36 | 1.59 |  |
| 25 | N Hoskins District (PNG) | I24S3c22 | S | 5°12' | E | 150°23' | 6 | 20/11/2017 | 29.76 | 34.41 | 0.39 | 23.60 | 8.41 | 1286 | 5189 | 20.97 | 1.50 | 1.23 | 1.85 |  |
| 26 | Helen Reef (Palau) | I25S1c1 | N | 2°57' | E | 131°48' | 3 | 22/12/2017 | 29.32 | 34.17 | 0.43 | 23.32 | 8.40 | 1109 | 4633 | 18.03 | 1.00 | 1.29 | 1.29 |  |
| 27 | Helen Reef (Palau) | I25S1c2 | N | 2°57' | E | 131°45' | 9 | 23/12/2017 | 29.32 | 34.17 | 0.43 | 23.21 | 8.40 | 1110 | 4691 | 18.02 | 1.13 | 1.26 | 1.42 |  |
| 28 | North Palau | I26S1c26 | N | 7°47' | E | 134°35' | 16 | 06/01/2018 | 29.16 | 33.99 | 0.39 | 23.9 | 8.44 | 1297 | 5050 | 20.93 | 1.00 | 1.55 | 1.55 |  |
| 29 | Koror (Palau) | I26S2 control 1 | N | 7°17' | E | 134°21' | 4 | 14/01/2018 | 29.20 | 33.98 | 0.40 | 23.21 | 8.40 | 1176 | 4968 | 19.06 | 1.40 | 1.51 | 2.11 |  |
| 30 | Koror (Palau) | I26S2 control 2 | N | 7°17' | E | 134°21' | 4 | 14/01/2018 | 29.20 | 33.98 | 0.39 | 22.76 | 8.37 | 1153 | 5142 | 18.71 | 1.50 | 1.52 | 2.28 |  |
| 31 | Green Island (Taiwan) | I28S3 | N | 22°39' | E | 121°29' | 3 | 24/03/2018 | 26.82 | 34.32 | 0.48 | 24.97 | 8.54 | 1175 | 4166 | 18.82 | 0.78 | 1.50 | 1.17 |  |
| 32 | Clipperton (France) | I31S3 | N | 10°18' | W | 109°12' | 10 | 09/08/2018 | 28.36 | 33.34 | 0.44 | 23.37 | 8.42 | 1066 | 4478 | 17.29 | 1.33 | 1.02 | 1.36 |  |
| 33 | Secas islands (Panama) | I32S1 | N | 7°57' | W | 82°03' | 3 | 25/08/2018 | 28.96 | 32.25 | 0.41 | 24.86 | 8.52 | 1289 | 4609 | 21.11 | 1.14 | 1.25 | 1.43 |  |
|  | ***Diploastrea* spp.** | |  |  |  |  |  |  |  |  |  |  |  |  |  |  |  |  |  |  |
| 20 | Noumea Lagoon (New Caledonia) | I21S2c17 | S | 22°33' | E | 166°26' | 15 | 26/09/2017 | 24.49 | 35.44 | 0.50 | 24.39 | 8.52 | 1110 | 4202 | 17.36 | 0.27 | 1.95 | 0.53 |  |
| 23 | Losuia, Tabungora Island (PNG) | I23S2 | S | 9°21' | E | 152°02' | 13 | 06/11/2017 | 28.69 | 34.63 | 0.44 | 23.36 | 8.41 | 1122 | 4648 | 18.02 | 0.50 | 1.52 | 0.76 |  |
| 24 | Kimbe, Hoskins District (PNG) | I24S2c3 | S | 5°05' | E | 150°12' | 8 | 18/11/2017 | 29.75 | 34.41 | 0.43 | 22.08 | 8.31 | 980 | 4715 | 15.91 | 0.50 | 1.48 | 0.74 |  |
| 25 | W Garua, N Hoskins District (PNG) | I24S3c23 | S | 5°19' | E | 150°07' | 7 | 02/12/2017 | 29.76 | 34.41 | 0.41 | 22.29 | 8.33 | 1069 | 5022 | 17.39 | 0.50 | 1.45 | 0.73 |  |
| 28 | North Palau | I26S1 | N | 7°47' | E | 134°35' | 16 | 06/01/2018 | 29.16 | 33.99 | 0.41 | 22.10 | 8.32 | 1023 | 4949 | 16.60 | 0.50 | 1.59 | 0.80 |  |
| 31 | Green Island (Taiwan) | I28S3 | N | 22°39' | E | 121°29' | 13 | 23/03/2018 | 26.82 | 34.32 | 0.46 | 22.95 | 8.41 | 1030 | 4538 | 16.37 | 0.60 | 1.58 | 0.95 |  |

**Table S2.** Physicochemical properties of seawater surrounding coral core sampling across the Pacific Ocean. Seawater temperature, salinity, and carbonate chemistry (pHsw, [CO32-]sw, [DIC]sw, Ωsw) for each studied site. "*Tara* seawater properties" correspond to discrete *in situ* measurements made during the *Tara*- Pacific expedition 1 and "6-yr integrated seawater properties " correspond to calculated data for the period 2010 – 2016 (see methods).

|  |  |  | |  | | **PUNCTUAL SEAWATER PROPERTIES** | | | | | | **INTEGRATED SEAWATER PROPERTIES** | | | | | |
| --- | --- | --- | --- | --- | --- | --- | --- | --- | --- | --- | --- | --- | --- | --- | --- | --- | --- |
|  | Location |  | Latitude |  | Longitude | SST | Salinity | pH | [CO_3_^2-^] | DIC | Ω | SST | Salinity | pH | [CO_3_^2-^] | DIC | Ω |
|  |  |  |  |  |  | °C |  |  | 𝜇mol·mol^-1^ | 𝜇mol·kg^-1^ |  | °C |  |  | 𝜇mol·kg^-1^ | 𝜇mol·kg^-1^ |  |
| 1 | Coiba (Panama) | N | 7°12' | W | 81°47' | 28.17 | 31.79 | 8.01 | 186 | 1822 | 3.06 | 28.59 | 31.52 | 8.03 | 195 | 1791 | 3.21 |
| 2 | Anakena, Isla de Pascua (Chile) | S | 27°04' | W | 109°19' | 20.84 | 35.72 | 8.06 | 201 | 2066 | 3.12 | 22.45 | 36.04 | 8.05 | 210 | 2069 | 3.26 |
| 3 | Motu Taka Rua, Isla de Pascua (Chile) | S | 27°04' | W | 109°19' | 20.86 | 36.04 | 8.07 | 206 | 2071 | 3.19 | 22.44 | 36.05 | 8.05 | 210 | 2070 | 3.26 |
| 4 | Tekava, Gambier (French Polynesia) | S | 23°09' | W | 134°50' | 22.84 | 35.87 | 8.07 | 215 | 2042 | 3.36 | 25.17 | 36.08 | 8.06 | 230 | 2042 | 3.61 |
| 5 | Moorea (French Polynesia) | S | 17°28' | W | 149°48' | 28.24 | 36.03 | 8.04 | 242 | 2024 | 3.87 | 27.77 | 35.91 | 8.06 | 248 | 2004 | 3.95 |
| 6 | Moorea (French Polynesia) | S | 17°29' | W | 149°45' | 28.25 | 36.03 | 8.05 | 248 | 2025 | 3.96 | 27.77 | 35.86 | 8.06 | 247 | 2002 | 3.94 |
| 7 | Aitutaki, Cook (New Zeland) | S | 18°50' | W | 159°48' | 27.29 | 35.43 | 8.05 | 234 | 1987 | 3.74 | 27.09 | 35.67 | 8.07 | 242 | 1996 | 3.85 |
| 8 | S Niue (New Zeland) | S | 19°06' | W | 169°54' | 28.18 | 34.80 | 8.06 | 243 | 2019 | 3.92 | 27.00 | 35.44 | 8.07 | 239 | 1985 | 3.81 |
| 9 | W Niue (New Zeland) | S | 18°59' | W | 169°54' | 28.31 | 34.73 | 8.05 | 232 | 1963 | 3.75 | 27.14 | 35.43 | 8.07 | 240 | 1983 | 3.83 |
| 10 | Upolu (Samoa) | S | 13°50' | W | 172°04' | 31.27 | 34.48 | 7.96 | 213 | 1956 | 3.49 | 28.81 | 35.17 | 8.06 | 243 | 1962 | 3.91 |
| 11 | CSR 11, Wallis (France) | S | 13°18' | W | 176°12' | 29.65 | 34.14 | 8.03 | 228 | 1926 | 3.72 | 28.97 | 35.06 | 8.05 | 242 | 1956 | 3.90 |
| 12 | Abaiang (Kiribati) | N | 01°43' | E | 172°58' | 28.68 | 35.13 | 8.00 | 217 | 1997 | 3.50 | 28.97 | 34.89 | 8.01 | 221 | 1977 | 3.58 |
| 13 | Pisinun, Chuuk (Micronesia) | N | 07°08' | E | 151°53' | 28.98 | 33.82 | 8.03 | 217 | 1913 | 3.54 | 29.23 | 34.09 | 8.05 | 232 | 1906 | 3.77 |
| 14 | Guam (USA) | N | 13°14' | E | 144°38' | 28.18 | 34.29 | 8.03 | 220 | 1940 | 3.56 | 28.92 | 34.46 | 8.05 | 234 | 1928 | 3.79 |
| 15 | Ogasawara (Japan) | N | 27°05' | E | 142°13' | 20.74 | 34.82 | 8.10 | 208 | 2018 | 3.24 | 24.30 | 34.78 | 8.07 | 214 | 1978 | 3.39 |
| 16 | Sesoko, Okinawa (Japan) | N | 26°38' | E | 127°51' | 21.71 | 34.96 | 8.08 | 205 | 2002 | 3.21 | 24.88 | 34.71 | 8.08 | 221 | 1963 | 3.51 |
| 17 | NW Fiji | S | 17°22' | E | 177°07' | 28.27 | 34.39 | 8.02 | 216 | 1938 | 3.49 | 28.03 | 35.08 | 8.07 | 241 | 1958 | 3.88 |
| 18 | Heron, S GBR (Australia) | S | 23°26' | E | 151°54' | 22.33 | 35.51 | 8.08 | 215 | 2029 | 3.36 | 24.43 | 35.39 | 8.09 | 230 | 1995 | 3.62 |
| 19 | SW Cockatoo Reef, GBR (Australia) | S | 20°46' | E | 150°53' | 23.18 | 35.44 | 8.06 | 214 | 2030 | 3.36 | 25.49 | 35.36 | 8.08 | 234 | 1987 | 3.70 |
| 20 | Noumea Lagoon (New Caledonia) | S | 22°32' | E | 166°26' | 22.87 | 35.84 | 8.08 | 219 | 2028 | 3.41 | 24.49 | 35.44 | 8.08 | 228 | 2001 | 3.59 |
| 21 | E Vangunu (Salomon) | S | 08°36' | E | 158° 11' | 29.84 | 34.00 | 8.01 | 214 | 1906 | 3.51 | 29.43 | 34.61 | 8.05 | 237 | 1934 | 3.85 |
| 22 | E Vangunu (Salomon) | S | 08°36' | E | 158° 11' | 29.33 | 34.09 | 8.04 | 228 | 1906 | 3.72 | 29.43 | 34.61 | 8.05 | 237 | 1934 | 3.85 |
| 23 | Losuia, Tabungora Island (PNG) | S | 9°21' | E | 152°02' | 29.23 | 34.34 | 8.05 | 234 | 1928 | 3.81 | 28.69 | 34.63 | 8.05 | 233 | 1940 | 3.77 |
| 24 | Kimbe, Hoskins District (PNG) | S | 5°05' | E | 150°12' | 30.84 | 34.13 | 8.04 | 232 | 1865 | 3.82 | 29.75 | 34.41 | 8.04 | 235 | 1924 | 3.82 |
| 25 | N Hoskins District (PNG) | S | 5°12' | E | 150°23' | 30.71 | 33.22 | 8.06 | 238 | 1888 | 3.94 | 29.76 | 34.41 | 8.04 | 234 | 1924 | 3.82 |
| 26 | Helen Reef (Palau) | N | 2°57' | E | 131°48' | 29.71 | 33.86 | 8.07 | 238 | 1859 | 3.89 | 29.32 | 34.17 | 8.05 | 231 | 1914 | 3.76 |
| 27 | Helen Reef (Palau) | N | 2°57' | E | 131°45' | 29.23 | 33.87 | 7.99 | 205 | 1911 | 3.34 | 29.32 | 34.17 | 8.04 | 230 | 1914 | 3.75 |
| 28 | North Palau | N | 7°47' | E | 134°35' | 29.11 | 33.81 | 8.05 | 226 | 1871 | 3.69 | 29.16 | 33.99 | 8.05 | 230 | 1902 | 3.74 |
| 29 | Koror (Palau) | N | 7°17' | E | 134°21' | 29.70 | 33.57 | 8.03 | 219 | 1872 | 3.59 | 29.20 | 33.98 | 8.05 | 230 | 1901 | 3.76 |
| 30 | Koror (Palau) | N | 7°17' | E | 134°21' | 29.70 | 33.57 | 8.03 | 219 | 1872 | 3.59 | 29.20 | 33.98 | 8.05 | 230 | 1901 | 3.76 |
| 31 | Green Island (Taiwan) | N | 22°39' | E | 121°29' | 26.04 | 34.18 | 8.05 | 216 | 1963 | 3.45 | 26.82 | 34.32 | 8.06 | 224 | 1933 | 3.60 |
| 32 | Clipperton (France) | N | 10°18' | W | 109°12' | 28.29 | 33.36 | 8.03 | 212 | 1902 | 3.45 | 28.36 | 33.34 | 8.02 | 206 | 1896 | 3.35 |
| 33 | Secas islands (Panama) | N | 7°57' | W | 82°03' | 29.04 | 31.04 | 8.03 | 192 | 1769 | 3.18 | 28.96 | 32.25 | 8.04 | 205 | 1823 | 3.38 |

**Table S3.** Linear extension rates of *Porites* core-tops (n = 33) measured on CT-scans according to two different methods of reading. Method A corresponds to the extension rates measured along the same 3 transects (Fig. S5) of density change measurements (values used in study) and method B corresponds to the extension rates measured on 3 different slices of coral core CT-scans (values confirming the first dataset within the 4 % analytical uncertainty).

|  | Location | Sample | Latitude | Longitude | Extension rate (cm·yr^-1^) | |
| --- | --- | --- | --- | --- | --- | --- |
|  |  |  |  |  | A | B |
|  | ***Porites* spp.** |  |  |  |  |  |
| 1 | Coiba (Panama) | I2S3c21 | 7°12' N | 81°47' W | 0.88 | 0.98 |
| 2 | Anakena. Isla de Pascua (Chile) | I4S1 | 27°04' S | 109°19' W | 1.00 | 1.14 |
| 3 | Motu Taka Rua. Isla de Pascua (Chile) | I5S4 | 27°04' S | 109°19' W | 1.33 | 1.22 |
| 4 | Tekava. Gambier (French Polynesia) | I6S2 | 23°09' S | 134°50' W | 0.83 | 0.96 |
| 5 | Moorea (French Polynesia) | I7S1c1 | 17°28' S | 149°48' W | 1.20 | 1.16 |
| 6 | Moorea (French Polynesia) | I7S3c2 | 17°29' S | 149°45' W | 1.25 | 1.18 |
| 7 | Aitutaki. Cook (New Zeland) | I8S1c3 | 18°50' S | 159°48' W | 1.00 | 1.04 |
| 8 | S Niue (New Zeland) | I9S1c4 | 19°06' S | 169°54' W | 1.20 | 1.23 |
| 9 | W Niue (New Zeland) | I9S2c5 | 18°59' S | 169°54' W | 0.86 | 0.89 |
| 10 | Upolu (Samoa) | I10S0c6 | 13°50' S | 172°04' W | 1.00 | 1.09 |
| 11 | CSR 11. Wallis (France) | I11S1c7 | 13°18' S | 176°12' W | 0.67 | 0.88 |
| 12 | Abaiang (Kiribati) | I13S3c8 | 01°43' N | 172°58' E | 1.33 | 1.38 |
| 13 | Pisinun. Chuuk (Micronesia) | I14S2c9 | 07°08' N | 151°53' E | 1.00 | 1.09 |
| 14 | Guam (USA) | I15S1c10 | 13°14' N | 144°38' E | 0.75 | 0.99 |
| 15 | Ogasawara (Japan) | I16S2c11 | 27°05' N | 142°13' E | 0.67 | 0.66 |
| 16 | Sesoko. Okinawa (Japan) | I17 | 26°38' N | 127°51' E | 1.14 | 1.14 |
| 17 | NW Fiji | I18S1 | 17°22' S | 177°07' E | 1.25 | 1.20 |
| 18 | Heron. S GBR (Australia) | I19S1c13 | 23°26' S | 151°54' E | 1.00 | 0.89 |
| 19 | SW Cockatoo Reef. GBR (Australia) | I19S3c14 | 20°46' S | 150°53' E | 1.50 | 1.57 |
| 20 | Noumea Lagoon (New Caledonia) | I21S2c16 | 22°32' S | 166°26' E | 1.00 | 1.06 |
| 21 | E Vangunu (Salomon) | I22S2c1 | 08°36' S | 158° 11' E | 1.50 | 1.67 |
| 22 | E Vangunu (Salomon) | I22S2c2 | 08°36' S | 158° 11' E | 1.29 | 1.25 |
| 23 | Losuia. Tabungora Island (PNG) | I23S2 | 9°21' S | 152°02' E | 1.00 | 0.99 |
| 24 | Kimbe. Hoskins District (PNG) | I24S2c2 | 5°05' S | 150°12' E | 1.17 | 1.17 |
| 25 | N Hoskins District (PNG) | I24S3c22 | 5°12' S | 150°23' E | 1.50 | 1.63 |
| 26 | Helen Reef (Palau) | I25S1c1 | 2°57' N | 131°48' E | 1.00 | 1.06 |
| 27 | Helen Reef (Palau) | I25S1c2 | 2°57' N | 131°45' E | 1.13 | 1.16 |
| 28 | North Palau | I26S1c26 | 7°47' N | 134°35' E | 1.00 | 0.94 |
| 29 | Koror (Palau) | I26S2 control 1 | 7°17' N | 134°21' E | 1.40 | 1.38 |
| 30 | Koror (Palau) | I26S2 control 2 | 7°17' N | 134°21' E | 1.50 | 1.25 |
| 31 | Green Island (Taiwan) | I28S3 | 22°39' N | 121°29' E | 0.78 | 0.81 |
| 32 | Clipperton (France) | I31S3 | 10°18' N | 109°12' W | 1.33 | 1.42 |
| 33 | Secas islands (Panama) | I32S1 | 7°57' N | 82°03' W | 1.14 | 1.18 |

**Table S4.** Statistical parameters of linear equations between coral skeleton geochemistry (δ^11^B, B/Ca), calcifying fluid carbonate chemistry (pH_cf_, DIC_cf_, [CO_3_^2-^]_cf_ and Ω_cf_), and SST (OiSSTv2). Graphs are plotted in Figures 3 and 4 of the main manuscript.

|  | **B/Ca** | **δ^11^B** | **DIC_cf_** | **pH_cf_** | **[CO_3_^2-^]_cf_** | **Ω_cf_** |
| --- | --- | --- | --- | --- | --- | --- |
| **P. n = 33** | -0.019 (0.002) SST + 0.98 (0.05) | -0.071 (0.061) SST + 25.84 (1.68) | 127.5 (23.5) SST + 1073 (648) | -0.015 (0.004) SST + 8.86 (0.11) | 29.11 (5.37) SST + 359.8 (148.4) | 0.59 (0.09) SST + 2.41 (2.40) |
|  | R^2^=0.76, P< 0.0001 | R^2^=0.04, P=0.25 | R^2^=0.49, P<0.0001 | R^2^= 0.30, P<0.001 | R^2^= 0.49, P<0.0001 | R^2^= 0.60, P<0.0001 |
| **P. n = 6** | -0.025 (0.003) SST + 1.13 (0.09) | -0.27 (0.07) SST + 31.68 (2.09) | 222.9 (37.7) SST - 1588 (1063) | -0.028 (0.006) SST + 9.25 (0.16) | 29.78 (9.70) SST + 373.0 (273.3) | 0.61 (0.16) SST + 2.43 (4.61) |
|  | R^2^=0.94, P=0.0013 | R^2^=0.77, P=0.02 | R^2^=0.90, P=0.004 | R^2^= 0.86, P=0.007 | R^2^= 0.70, P=0.04 | R^2^= 0.77, P<0.02 |
| **D. n = 6** | -0.016 (0.002) SST + 0.88 (0.07) | -0.39 (0.10) SST + 33.77 (2.80) | 129.2 (30.3) SST - 1047 (853.7) | -0.036 (0.006) SST + 9.41 (0.18) | -12.18 (11.67) SST + 1398.0 (328.8) | -0.07 (0.18) SST + 18.88 (5.20) |
|  | R^2^=0.91, P=0.003 | R^2^=0.79, P=0.02 | R^2^=0.82, P=0.01 | R^2^=0.89, P=0.005 | R^2^= 0.21, P=0.36 | R^2^= 0.82, P=0.01 |


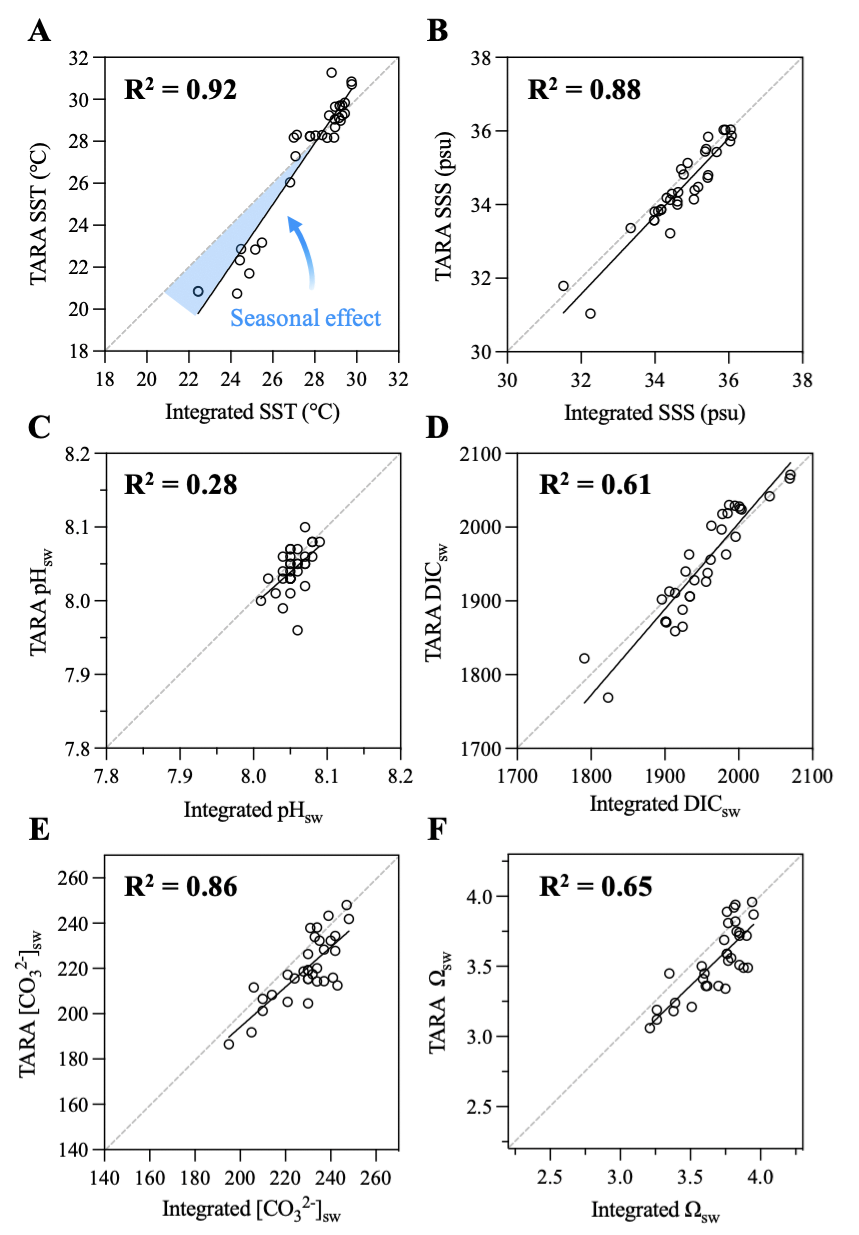


**Figure S1.** Linear regressions between ‘*Tara* discrete’ and ‘6-yr integrated’ seawater properties. **(A)** Sea surface temperature in °C, **(B)** Sea surface salinity **(C)** pH, **(D)** DIC in μmol·kg-1, **(E)** [CO32-] in μmol·kg-1 and **(F)** Ω.  *Tara* discrete values correspond to *in situ* measurements from the *Tara*-Pacific expedition near the drilling sites (a few meters) 1. ‘6yr-Integrated’ values are exported from databases and correspond to regional grids (from 0.25x0.25° to 2x2°) over the period 2010 – 2016. The gray dotted lines are 1:1. All regressions are significantly positive (P < 0.0001).

Here, strong and significant correlation is observed between the discrete SST measured during the *Tara*-Pacific expedition and AVHRR-SST (R^2^ = 0.92, P < 0.001). It is noted that *Tara*’s SST data in the highest tropical latitudes are strongly impacted by the seasonal effect. Indeed, comparing the SST *in situ* with the monthly AVHRR-SST at the time (month) of sampling, the SST is similar within the errors (ΔT < 0.5 °C, where ΔT represents the discrepancies between punctual in situ SST and the respective monthly AVHRR-SST). The study sites, characterized by seasonality are those where temperatures reach the lowest values (integrated SST = 22 – 26 °C) and deviate from the ideal 1:1 line, as the integrated values are free of it (Fig. S1-A). In contrast, in equatorial and subtropical regions, where seasonal variability is less (or not at all) noticeable, the *Tara*’s temperatures align well with the integrated values and with the ideal 1:1 line (SST = 27 – 31 °C, Fig. S1-A). The seasonal effect is also illustrated by the wider temperature range of the measured data (18 - 32 °C for *Tara* points) compared to the regional 6-year integrated data (22 – 30 °C for databases).

There is a strong and significant correlation between the discrete SST measured during the *Tara*-Pacific expedition and the AVHRR SST (R^2^ = 0.92, P < 0.001). It should be noted that the *Tara* SST data in the highest tropical latitudes are strongly influenced by the seasonal effect. Indeed, comparing the *in situ* SST with the monthly AVHRR SST at the time (month) of sampling, the SST is similar within the errors (ΔT < 0.5 °C, where ΔT represents the discrepancies between the punctual in situ SST and the corresponding monthly AVHRR SST). The study sites characterized by seasonality are those where temperatures reach the lowest values (integrated SST = 22 – 26 °C) and deviate from the ideal 1:1 line, as the integrated values are free of it (Fig. S1-A). In contrast, in equatorial and subtropical regions, where the seasonal variability is less (or not at all) pronounced, the *Tara* temperatures agree well with the integrated values and with the ideal 1:1 line (SST = 27 – 31 °C, Fig. S1-A). The seasonal effect is also illustrated by the wider temperature range of the measured data (18 – 32 °C for *Tara* points) compared to the regional 6-year integrated data (22 – 30 °C for databases).

**Figure S2.** Spearman rank correlations of seawater parameters, coral skeleton, calcifying fluid, and growth variables. Color indicates strength and direction of correlation (red, positive; blue, negative). The numerical values indicate the (**A**) Spearman R's and (**B**) *p*-values, which are significant in bold (P < 0.05, significance increases when |R| is close to 1).

In order to compare all variables between them, only the genus *Porites* was considered for the correlation matrix as the number of colonies (n = 33) is much more representative of the Pacific Ocean scale than that of the genus *Diploastrea* (n = 6). The dataset for seawater temperature, salinity, and carbonate chemistry considered in this section was compiled from measured, satellite, and modelled data (previously referred to as ‘integrated 6-yr’ values) for the period 2010-2016. The B/Ca and δ11B values measured in coral skeletons have been used to reconstruct the composition of their calcifying fluids (pHcf, [CO32-]cf, DICcf, Ωcf). This matrix gives calculated R coefficients for each pair of variables, where R indicates the direction and robustness of the correlations and P indicates the two-tailed significance with a 95% confidence interval.

Only major correlations are described for *Porites*. B/Ca is negatively correlated with SST (P < 0.001) but positively correlated with SSS, pH_sw_ and DIC_sw_ (P < 0.001). These results suggest a strong dependence of B/Ca measured in coral skeletons on ambient seawater variations of SST, SSS, DIC_sw_ and pH_sw_. Here, no correlation between δ^11^B and seawater properties was observed (P > 0.05), suggesting that environmental factors other than SST or carbonate chemistry properties (i.e. the so-called "vital effects") influence the boron isotopic composition in the coral skeleton. For temperature, our result is consistent with previous studies showing no effect of temperature on δ^11^B ^2,3^. However, this observation is inconsistent with previous studies on cultured *Acropora* spp. that found a positive relationship between temperature and δ^11^B ^4^.

*Porites* δ11B-derived pHcf shows a significant negative relationship with temperature (R = -0.63, P < 0.05) and a positive relationship with DICsw (R = 0.41, P < 0.05). However, pHcf at the site of calcification was not consistently dependent on seawater pHsw, [CO32-]sw or Ωsw (P > 0.05). Among the variables studied, temperature explained the most variance in coral pHcf while seawater pHsw, [CO32-]sw, DICsw or Ωsw did not explain any additional variable in pHcf variability. These results are consistent with Ross et al. (2019) 5, who showed similar negative relationships between pHcf and SST at the seasonal level and no correlation between pHcf and pHsw.

The other parameters of the coral calcifying fluid show similar correlations with seawater properties. Indeed, [CO32-]cf, DICcf and Ωcf show strong positive correlations with SST (R = 0.59, 0.71 and 0.69, respectively, P < 0.05) and negative correlations with salinity (R = -0.36, -0.45 and -0.48, respectively, P < 0.05), pHsw (R = -0.41, -0.39 and -0.51, respectively, P < 0.05), and DICsw (R = -0.42, -0.52 and -0.54, respectively, P < 0.05). Only [CO32-]cf and Ωcf are positively correlated with seawater Ωsw (R = 0.41 and 0.36, respectively, P < 0.05).

Finally, this study assessed the correlations between coral growth parameters, calcifying fluid, and ambient seawater properties. Therefore, linear extension rates display a negative relationship with δ11B and pHcf (R = -0.37 and -0.36, respectively, P < 0.05). Skeletal density is not significantly dependent on either seawater or calcifying fluid parameters (P > 0.05). However, other potential relationships between [CO32-]cf, or Ωcf and density, and between DICcf and the linear extension can also be identified, in agreement with previous observations in the literature 6. Calcification rates increase significantly with SST and DICcf (R = 0.37 and 0.35, respectively, P < 0.05). Although SST and cf carbonate chemistry partially explain the variability in skeletal growth/calcification of *Porites*.

**Figure S3.** Relationships between the parameters of the coral calcifying fluid. (**A**) Aragonite saturation state Ωcf *vs*. SST, and (**B**) pHcf *vs*. DICcf/DICsw of massive *Porites* corals. Black dots correspond to *Porites* colonies from this study (n = 33). The colored dots represent the seasonal records of four *Porites* colonies (CB-1, CB-2, D2, and D3) from Australia 7.

The inverse correlation between pHcf and DICcf / DICsw (R2 =0.57, P < 0.001, Fig. S4-B) in the *Porites* of this study highlights the important role of DIC, both in seawater and calcifying fluid, in regulating the pH_cf_ of *Porites* colonies over a range of temperatures observed in the tropical Pacific (22 – 30°C). These two correlations observed at the Pacific Ocean scale are comparable to those found by McCulloch et al. (2017)^7^, who studied four Australian *Porites* colonies on a seasonal scale over a similar range of SST changes. At the Pacific Ocean scale and at annual resolution, this supports the theory that corals exert a strong biological control that allows the regulation of CF carbonate chemistry in favor of skeletal secretion, primarily controlled by temperature, but also by dissolved inorganic carbon and/or pH properties.


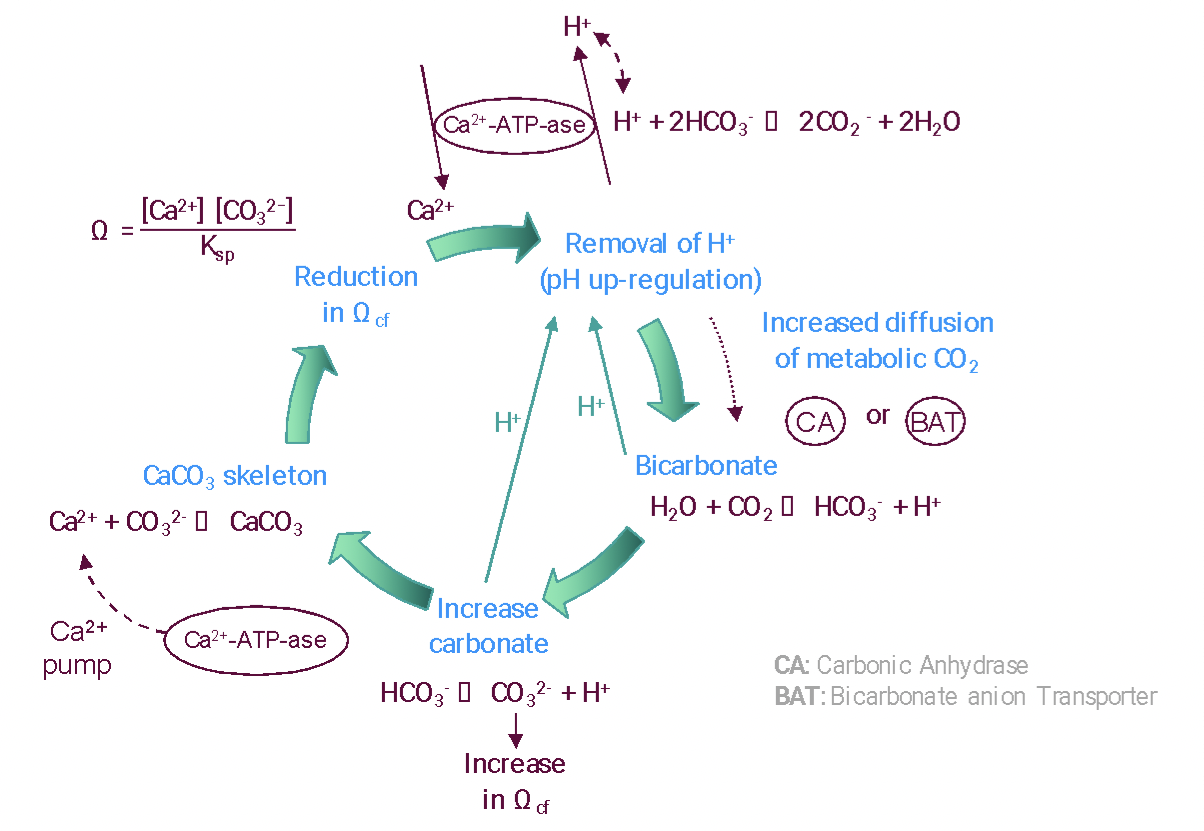


**Figure S4.** Schematic model of the calcification process in corals occurring within the extracellular calcifying fluid located between the subcalicoblastic cells and the growing skeleton (modified from Ross et al., 2018, 2019) 5,8. Membrane-bound isoform of Carbonic Anhydrase (CA) and of Bicarbonate Anion Transporter (BAT).

Similar B/Ca ratios are measured in *Porites* and *Diploastrea* corals with increasing temperatures (Fig. 3-A). The δ11B values of *Diploastrea* are systematically and significantly lower than those of *Porites* although grown at the same site (Fig. 3-B, n = 6, full red and blue dots respectively). This result suggests that factors other than temperature or pH_sw_ (i.e. so-called "vital effects") influence boron isotopic composition in massive coral skeletons. However, at the 6 sites studied, δ11B values decrease with temperature in both genera, with a more pronounced trend in *Diploastrea*. B/Ca is negatively correlated with seawater temperature in *Diploastrea*. In *Porites*, all cf parameters studied (i.e. pHcf, [CO32-]cf, DICcf, Ωcf) are significantly correlated with SST, whereas in *Diploastrea,* only pHcf and DICcf are significantly correlated with temperature (P < 0.05). For both *Porites* and *Diploastrea,* pHcf and DICcf show similar relationships with temperature, negative and positive respectively (Fig. 3-C). For both genera, DICcf increases significantly with temperature (Fig. 3-D). At temperatures above ~ 27 °C, *Diploastrea* [CO32-]cf and Ωcf values are systematically lower than those of *Porites* and inverse relationships between [CO32-]cf or Ωcf and temperature are observed in *Porites* and *Diploastrea* (Fig. 3-E, F). Indeed, [CO32-]cf and Ωcf increase significantly with temperature in *Porites*, whereas [CO32-]cf and Ωcf are not significantly correlated with seawater temperature in *Diploastrea*. In comparison, at seasonal and intra-colonial levels, four branching corals showed a decrease in [CO32-]cf with temperature in the study of Ross et al. (2019).


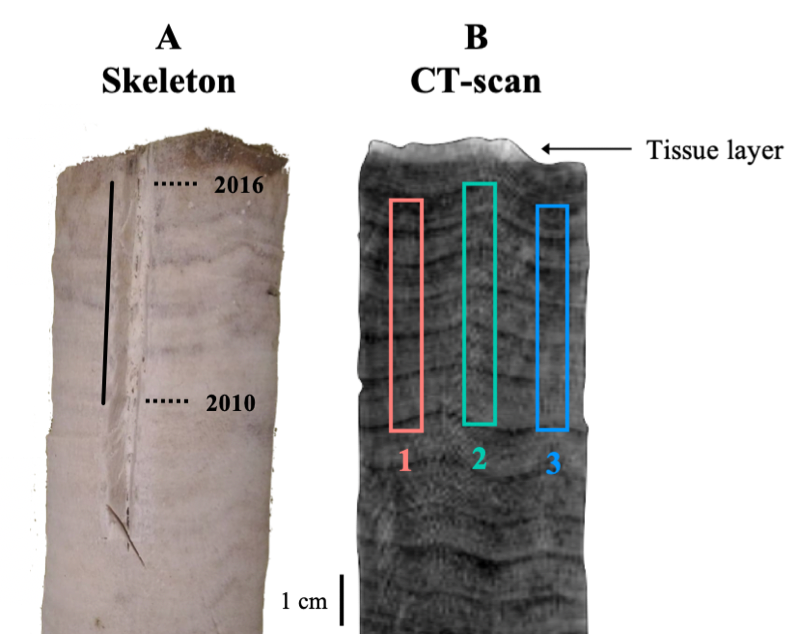


**Figure S5.** Bulk sampling strategy and density measurements for the period 2010-2016. (**A**) Image of the coral skeleton of the *Porites* colony from Fiji (I18S1), (**B**) Relative CT-scan image of a slice of the colony in negative where density was measured along 3 growth axes of the upper- most section (2010 – 2016) of the colony.

**References**

1. Douville, E. et *al.* Seawater carbonate chemistry dataset collected during the Tara Pacific Expedition 2016-2018. *PANGAEA*, https://doi.org/10.1594/PANGAEA.9444202.
2. Hemming, N. G. & Hanson, G. N. Boron isotopic composition and concentration in modern marine carbonates. *Geochim. Cosmochim. Acta* **56**, 537–543 (1992).
3. Reynaud, S., Hemming, N. G., Juillet-Leclerc, A. & Gattuso, J.-P. Effect of *p*CO2 and temperature on the boron isotopic composition of the zooxanthellate coral *Acropora* sp. *Coral Reefs* **23**, 539–546 (2004).
4. Dissard, D. *et al*. Light and temperature effects on δ11B and B/Ca ratios of the zooxanthellate coral *Acropora* sp.: results from culturing experiments. *Biogeosciences* **9**, 4589–4605 (2012).
5. Ross, C. L. DeCarlo, T. M. & McCulloch, M. T. Environmental and physiochemical controls on coral calcification along a latitudinal temperature gradient in Western Australia. *Glob. Change Biol.* **25**, 431–447 (2019).
6. Mollica, N. R. *et al.* Ocean acidification affects coral growth by reducing skeletal density. *Proc. Natl. Acad. Sci.* **115**, 1754–1759 (2018).
7. McCulloch, M. T., D’Olivo, J. P., Falter, J., Holcomb, M. & Trotter, J. A. Coral calcification in a changing World and the interactive dynamics of pH and DIC upregulation. *Nat. Commun.* **8**, 15686 (2017).
8. Ross, C., Schoepf, V., Decarlo, T. & McCulloch, M. T. Mechanisms and seasonal drivers of calcification in the temperate coral *Turbinaria reniformis* at its latitudinal limits. *Proc. Royal Soc. B.* **285**. 20180215 (2018).
